# The landscape of musical care during the beginning of life in the United Kingdom: a mixed-methods survey study

**DOI:** 10.1186/s12906-025-05014-6

**Published:** 2025-10-16

**Authors:** Neta Spiro, Katie Rose M. Sanfilippo, Caitlin Shaughnessy, Mark Rowles, Elizabeth Coombes, Rosie Perkins, Emily Tredget

**Affiliations:** 1 Centre for Performance Science, Royal College of Music, London, UK; 2 Faculty of Medicine, Imperial College London, London, UK; 3 School of Health and Medical Sciences City St George’s, University of London, London, UK; 4 Faculty of Life Sciences and Education, University of South Wales, Newport, UK; 5 Happity, London, UK

**Keywords:** Infancy, Musical Care, Music, Parental, Perinatal, Postnatal, Pregnancy, Stepped care, Survey

## Abstract

**Background:**

The first 1001 days of life are a critical time in children’s development and can be challenging for parents and caregivers. Some families in the United Kingdom (UK) are not getting the support they need. Research indicates that musical care – the role of music in supporting any aspect of people’s developmental or health needs – can support families during, what we term, the beginning of life: pregnancy to two years of age. Musical care activities can take place in health and community settings and include music making, music listening, and music therapy. We must describe and understand the patterns of use of musical care activities during the beginning of life in the UK to capitalise on the potential of musical care to support families.

**Methods:**

This article explores, from parents’ and musical care providers’ perspectives, (1) participation and provision of musical care activities, (2) descriptions and experiences of musical care activities, and (3) motivations for, deterrents from, and perceived outcomes of participation in musical care activities. Data from two co-developed cross-sectional surveys for parents/caregivers (*N* = 578) and providers (*N* = 50) was analysed using descriptive statistics and thematic analysis.

**Results:**

Most parent/caregivers had participated in at least one musical care activity (83%). The most attended activity was play and development groups for babies that involve some music. Following our thematic analysis, personal preference, experiential and practical factors, recommendation by healthcare providers, and expectation of benefit were identified as motivators while deterrents included challenges in resources and logistics, and lack of inclusivity and diversity. Parents/caregivers perceived both positive and negative outcomes of attending musical care activities. Most providers had not had specific training and for many this work was not their primary income source.

**Conclusions:**

There is a wide range of musical care activities during the beginning of life in the UK. Reasons for attending them range from those specific to music and its care potential to seeing them as leisure activities. The findings have implications for the flexibility and role that musical care activities can play during the beginning of life and call for investigation into how musical care activities may be integrated into care.

**Supplementary Information:**

The online version contains supplementary material available at 10.1186/s12906-025-05014-6.

## Background

The first 1001 days of life are critical in children’s development. Development in this period is fast and in many areas: expressive and receptive language development, gross and fine motor control, social and emotional development, and self-regulation [1]. This is also a time when infants are particularly vulnerable [2]. At the same time, this period can be a challenging time for parents and caregivers with implications for their own physical and mental health [3]. The vulnerabilities of this critical period can be exacerbated when families encounter mental health challenges and inequities associated with social determinants of health [4–7]. For example, postnatal depression can negatively impact mother–infant bonding [3] and children's social-emotional development [8]. Early intervention, such as that provided by health visitors, has been found to be effective in improving outcomes for vulnerable children and their families [9, 10]. While it is important to support families – both the parents and their infants – during this period in a variety of ways, a recent report suggested that at least some families in the UK are not getting the support they need [2].

Many families face barriers to accessing health care. These are often associated with socioeconomic factors including gender [11], economic and geographic factors [12], and ethnicity [13]. There can be further barriers particular to this life stage. For example, though postnatal depression treatments exist, there are barriers to accessing care ranging from waiting lists for treatment [14] to reluctance to or lack of clarity about when and how to disclose concerns [15] and therefore seek help. Fear of stigma and judgement can also isolate parents during this time, particularly for women from disadvantaged communities [16].

Mounting research in the areas of music and health, music therapy, and music education suggests that engagement with music can support infants and their caregivers [17–23] and has the potential to fill gaps in existing provision [24–26]. The wide variety of possible musical activities offers a tantalising possibility of supporting families in ways that are appropriate for them at this life stage. However, there is little data about why families at this life stage choose to engage or not to engage in music activities and how they experience them. Addressing this gap is a first step in informing how local and national systems can integrate musical care to support families during the beginning of life across the UK.

We begin with broad and inclusive views of what we term the ‘beginning of life’ and ‘musical care’. We define the beginning of life as starting in pregnancy and continuing until the infants are two years old. Temporally, this aligns with the first 1001 days. This time period is acknowledged in research as important as it focuses on both the antenatal and the postnatal period considering both infant development and parents’ mental health [27]. The term the beginning of life also brings with it the importance of the familial/caregiving unit. Much research about the first 1001 days has focussed on mothers and their infants [28, 29]. However, others are involved in caregiving and are impacted by it. For example, research has suggested that fathers have an increased risk of mental health conditions during the perinatal period [28]. This is associated with maternal depression and can impact fathers’ ability to support their partners [28]. Additionally, 40% of grandparents in the UK over the age of 50 provided childcare in 2017 [30] and research has suggested higher burden of care on grandmothers (compared to grandfathers) and a need for more support for these caregivers [31, 32]. From our life stage perspective, we acknowledge the multi-facetted nature of families’ experiences during the beginning of life. Focus can be on different people – infants, their caregivers (acknowledging the full spectrum of diverse family arrangements) – and the relationships between them. Focus can also be on different outcomes including supporting infant wellbeing and development, parental/caregiver mental and social wellbeing, and bonding between infants and their parents/caregivers.

Musical care is defined as “the role of music – music listening as well as music-making – in supporting any aspect of people’s developmental or health needs, for example physical and mental health, cognitive and behavioural development, and interpersonal relationships” ([33], pp. 2–3). During the beginning of life this includes supporting the health and well-being of infants, caregivers, and the relationship between them. Musical care activities can happen in health and community settings (e.g., clinical contexts, schools, children’s centres, family hubs), may be administered by a variety of providers (e.g., health and social care providers, third sector organistions, educational providers, private organistions, and individuals), and can include a range of activities (e.g., music making, music listening, and music therapy).

Musical care activities include different types of music-based approaches. MacDonald describes five overlapping music, health, and well-being activities and practices: music therapy, community music, music medicine, music education, and everyday uses of music [34]. Music therapy emphasises the therapeutic relationship between a trained and licenced music therapist and the client(s) who together work towards specific therapeutic goals. Community music provides opportunities for musical engagement within local communities. In music medicine “prescribed music” is used with a specific health outcome in mind. Music education focuses on developing music skills and is often embedded in school settings. Everyday uses of music, while not a distinct practice, includes the receptive and participatory ways people engage with music (e.g., [35]). Some activities and practices within these five categories can target specific needs of an individual or group (e.g., mothers with postnatal depression, [36]) and some can address broader issues of, for example, mental health and wellbeing of parents attending a community choir. While recognizing the risk of conflating different practices or disciplines, in this article we conceptualise these together as musical care activities that draw on distinct evidence bases, practices, and disciplines [33].

Musical care research focussed on the beginning of life has suggested that music can support parental mental and social wellbeing [17], increase bonding and connection between parent and infant [37], and support infants’ wellbeing and development [25, 26]. For example, during pregnancy and birth, research has suggested that music listening can reduce anxiety symptoms in pregnancy as well as labour anxiety and pain [38]. In cases of postnatal depression, singing classes were found to speed up recovery from symptoms [36] and online songwriting groups were found to reduce loneliness and improve social connectedness for mothers [39, 40].

After birth, bedside music therapy can support maternal-infant bonding [37]. In families in which the mother is experiencing depression, research has suggested that interaction coaching in music therapy, where a music therapist models infant-directed singing to the mother, can help support the mother and the interactions with her infant [41–43]. Music therapy has been shown to support preterm infants and their parents in the neonatal intensive care unit [44–46], as well as supporting the whole family through family-based group approaches [47]. In terms of infants’ development, infants who had had 6 months of weekly active participatory music sessions beginning at 6 months of age showed “superior development of prelinguistic communicative gestures and social behaviour” compared to infants who had had passive music sessions [48].

Much of the work in these formal activities of musical care is based on, and is closely connected to, everyday musical activities, such as singing songs, listening to music, and infant-directed speech and singing [49, 50]. Evidence suggests that these everyday activities can positively affect infant emotional and arousal self-regulation, infant-parent bonding [51, 52] and infant pro-social behaviours [53]. These positive outcomes are understood to be connected to the repetition and temporal predictability of music [54], caregivers’ highly stereotyped and emotive performances [55, 56], caregivers’ use of highly familiar musical materials [57], and their multimodal music-making being attuned to cultural norms and infants’ momentary needs [58].

As evidenced by these examples, musical care activities during this life stage are varied. They include everyday musical care practices that can happen at home (such as singing and/or playing with musical instruments or toys) as well as formal musical care activities, including a range of musical practices that are offered in health sector and community settings (such as music therapy and community music). This variety of practices, with different access routes, taking place in different settings, and with potential for adaptation to specific contexts, makes musical care activities a tantalising network of practices that could be further developed to support families during the beginning of life. However, it remains unclear how families and providers see this work; their experiences of it; and their motivations for participating in or leading this work. Therefore, the overarching aim of this article is to understand and describe the patterns of use of musical care activities during the beginning of life in the UK.

## Methods

### Aim

This article will describe, from the perspectives of both parents/caregivers and providers their (1) participation and provision of musical care activities, (2) descriptions and experiences of musical care activities, and (3) their motivations for, deterrents from, and perceived outcomes of participation in musical care activities.

### Design

This study was completed in two phases: A survey co-development phase that included an online form and a preparatory focus group consultation with parents/caregivers and other relevant stakeholders (Phase 1), and two cross-sectional surveys – one for parents/caregivers and one for providers (Phase 2). The Consensus-Based Checklist for Reporting of Survey Studies [59] was used in preparing this article (Additional File 1). Ethical approval for the whole project was given by the Conservatoires UK Research Ethics Committee on 18th February 2022, ID: CUK/SF/2021–22/8.

#### Phase 1: Survey development consultation

The main aims of the survey development consultation phase were to ensure that the surveys address the key open questions about how parents/caregivers and providers experience musical care activities and what the barriers and opportunities might be. Through two, one-hour focus groups held on the 23rd of March 2022 or an online feedback form (using google forms) we asked the stakeholders specific questions about (1) terminology, phrasing, and answer options of specific questions, (2) the scope of musical care activities that should be included, and (3) our plans for survey dissemination (The focus group prompts and feedback form can be found in Additional Files 2 and 3).

##### Recruitment and participants

Recruitment took place through three routes; the professional networks of the team, particularly that of ET through her app Happity, and contacting organisations who work in the areas of early years or musical care. Of 16 participants, 9 took part in the focus groups and 7 used the feedback form. They included service users, experts in public health policy, experts in health care implementation, and musical care providers (see Table 1). Participants were each offered an online gift voucher (£20) in recognition of their time.

**Table 1:** Areas of expertise and geographic region of consultation participants

	**Total**	**Focus group**	**Form**
**Group**			
Parents	5	3*	2
Musical care professionals	7	5	2**
*Arts therapies professionals*	1	0	1
*Mental health professionals*	3	0	3**
*Medical professional*	2	2	0
*Lecturer*	2	2*	0
**Region**			
Northern Ireland	2	1	1
South Wales	5	3	2
South East England	4	3	1
East Midlands	1	0	1
East of England	1	0	1
London	3	2	1

^*^ one focus group participant was a parent and a lecturer, and another was a medical professional and lecturer

^**^ one form respondent was a mental health professional and a musical care professional

During this process we finalised several aspects of the surveys including: (1) The range of activities that we would list as part of musical care activities. For example, we added “Play and development groups” and included the option to add other activities; (2) The phrasing of the questions. For example, we collectively reached the wording “Could you tell us what it was like for you?” as a prompt for asking participants to describe their experiences of the musical care activity; (3) The key areas we should ask about. For example, how activities are advertised, evaluated, and accessed, and issues of diversity and inclusion; (4) Organisations that we could contact to disseminate the survey; and (5) Identification of the organisations to list in the survey for participants who felt they needed more support.

#### Phase 2: Two online cross-sectional surveys

##### Survey content

In order to address the same topics from two perspectives, two cross-sectional surveys were developed – one for parents/caregivers and one for providers. They were in line with each other as much as possible. Both included a mix of question response types including both open and closed questions. The parent/caregiver survey was aimed towards parents/caregivers who were expecting a baby or had had a baby during the last five years. The provider survey was aimed towards providers (e.g., music therapists, play group facilitators, community musicians) who over the last five years had run or were currently running musical care activities for families who were expecting a baby or had babies up to two years of age. We included the five-year period in recognition of the changes in in-person musical care activity provision during the COVID-19 pandemic.

*Parent/Caregiver survey*

The parent/caregiver survey included 7 sections:

1. Welcome and informed consent process
2. Familiarity with and participation in musical care activities
3. Experience of a specific musical care activity
4. Ideas for ways to increase the availability of musical care activities
5. Demographic questions
6. Optional contact details and entry into a draw for a voucher (for those recruited through social media)
7. Sources of support (for the full survey see Additional File 4).

Sections 2 and 4 were split into three subsections covering three target groups for the musical care activity: parents during pregnancy, parents during their child’s first two years of life, and babies during their first two years of life. This split was designed to recognise the difference in the focus of the activity in terms of who they are designed for.

Section 2 was about respondents’ awareness and participation in musical care activities. Through a co-developed list, we asked about formal musical care activities (e.g., Music classes/groups for expectant parent(s)/caregiver(s), Play and development groups that involve some music for parent(s)/caregiver(s), and Baby massage that involves some music). We also asked about informal activities (such as Informally singing or making music and Listening to music (on a personal device/at home)). The responses to the informal activities questions are not reported in this paper. Respondents were asked to select from the co-developed list the formal musical care activities that they had heard about and/or to add any activities not listed. For each option they selected, they were asked if they had participated in the activity and how they had heard about it. If they had not participated, they were asked if it had been available to them. All respondents were asked if they would have liked to participate in (more) formal musical care activities. If they answered yes, using closed and open questions, they were asked about the barriers that had stood in their way and what would support them in participating. At the end of this section, through closed questions, all participants were asked about what aspects were most important to them in participating in a formal musical care activity (e.g., Inclusivity and feeling welcome, Location (e.g., distance from home), or Training/credentials of group leader).

In Section 3 we asked the respondents about their experience of one example of a musical care activity in which they had participated (if applicable) that was the most memorable to them (whether for positive or negative reasons). Using open questions, we asked them to describe the activity, what it was like for them, why they decided to take part, how it was described to them, whether they gained skills or knowledge, to share online information about the activity (e.g., a website), and what they thought could increase the diversity of the group. Closed questions about the activity included when and how frequently they attended the activity, the cost, and how diverse they felt the activity was. The questions also asked about how they heard of the activity, how it had affected them (e.g., that they were more or less worried or that they agreed or disagreed that they met new friends), and whether they would recommend the activity to others.

Section 4 asked respondents to rank what types of formal musical care activities they would like to see offered more and an open question about what barriers they think there are to accessing musical care activities.

Section 5 included demographic information including region, age, gender, relationship and living status, disability status, ethnicity, household income, education, number of children, whether they were currently expecting a baby, and whether they were on parental leave. Musical experience was asked about in a single question about whether they work in music professionally and using the General Musical Sophistication subscale from the Gold MSI (alpha = 0.93; [54]).

*Provider survey*

The provider survey included 6 sections (Additional File 5). Sections 1, 5 and 6 mirrored Sections 1, 6 and 7 of the parent/caregiver survey. Section 2 focussed on the formal musical care activities they had delivered during the last five years for parents/caregivers during pregnancy, parents during their child’s first two years of life, and babies during their first two years of life. The section began with closed questions to select all activities they had run and ended with a series of questions about a specific example. These questions asked providers to describe the activity, its aim, who was invited, demographic information about who took part, how it was advertised, evaluated and funded, and whether they had had any training for this work. Section 3 examined ideas for ways to increase the availability of musical care activities. It began with open questions about the barriers and enablers in delivering musical care activities. Questions then asked about opportunities for funding and continuing education, workforce capacity building, and relevant stakeholders to support scale-up. They were also asked about whether and how they had tried to address inclusivity and diversity challenges. Section 4 included demographic information including region, age, gender, disability status, ethnicity, household income, education and training specific to this work. They were asked about how they describe their musical care work and if it is their main source of income.

### Data collection

The research team (which includes parents and musical care practitioners) piloted both surveys primarily to check clarity and survey logic. The parent/caregiver survey was open 21st June 2022 to 18th July 2022. The provider survey was open 8th of June 2022 to 27th of July 2022.

### Recruitment

#### Parents/Caregivers

Recruitment for the parent/caregiver survey was conducted through two methods: (1) social media through purposive sampling informed by our professional networks which had been expanded in Phase 1 and (2) Prolific, an online platform that distributes surveys and pays participants that are registered on their platform. Respondents who have registered provide key demographic information that can be used as part of pre-screening for recruitment; respondents are only invited to surveys if they match requested demographics. The platform also has a tool to ensure no unauthorised access and block multiple participation by the same respondents. There is also a quality assessment that includes screening for bots and assessing engagement with the survey. Prolific also has recommended payment rates based on time taken to complete the surveys. The survey was designed to take 20 min. We paid £8 per hour which meant that respondents received £2.67. Respondents recruited through social media were offered the opportunity to enter a draw for a £20 Amazon gift voucher. The survey was created in Qualtrics which includes bot detection and overall quality checks.

We aimed to recruit respondents from a range of ethnic backgrounds and from the regions around the UK broadly in Line with the proportions of ethnicity and region as represented in the 2021 census. As we were interested representation across the UK, we included a slightly greater proportion of people living in Wales, Scotland, and Northern Ireland. We recruited using an iterative process across two stages. Inclusion criteria during the initial stage were adults aged 18 years and older who lived in the UK and had had at least one child in the last 5 years. 381 respondents started the survey as part of this phase (74 through social media Links and 307 through Prolific). In the second stage, based on our initial participant characteristics, we limited recruitment to participants with specific geographic and income demographic characteristics to line up with national proportions (*n* = 236) using Prolific’s pre-screening process. For example, we limited recruitment to particular regions (e.g., people living in the East of England, Northern Ireland, and Wales) or to those with particular income ranges (e.g., income of £19,999 or less) according to quotas that aligned with national distributions. This provided an initial data set of 617 responses. The data set was then analysed manually to remove ineligible responses (e.g., incomplete responses, nonsense words, *n* = *39*), leaving a data set of *N* = 578.

#### Providers

Using purposive sampling, recruitment for the providers survey was through three routes: (1) the professional networks of the team, particularly that of ET through her app Happity, (2) contacting organisations who work in the areas of early years or musical care by email, or (3) through social media. The inclusion criteria were adults aged 18 years and above, currently living and working in the UK, currently or in the last five years working in musical care with people expecting a baby and/or during their baby’s/babies’ first two years. Respondents were offered the opportunity to enter a draw for a £20 Amazon gift voucher. The survey was created in Qualtrics which includes bot detection and overall quality checks.

## Survey analysis

Responses to numeric and closed questions were summarised using descriptive statistics in Excel [60]. Analysis was undertaken for the open questions using Dedoose [61]. Starting with the parent/caregiver survey, two authors (CS and MR) carried out an initial inductive process of coding and analysis using thematic analysis [62]. This offered a way to systematically identify and organise responses whilst providing understanding of wider themes across the data set. Each respondent’s survey was analysed in its totality. This approach allowed for the analysis of survey data to include either descriptive or reflexive interpretation depending on the depth of response [63]. The briefer responses were grouped into descriptive codes, but more detailed codes and subthemes were developed for the more expansive responses. The provider survey was then analysed using the coding framework of the parent/caregiver survey where relevant. In addition, an initial inductive process of coding and analysis using thematic analysis was carried out for responses on new areas (such as “providers’ motivations”).

CS and MR independently coded the responses. At equal points during the analysis, CS and MR met to discuss and cross-check themes and interpretations, discuss any disagreements and referred to NS and KRS if needed. For example, the coders encountered differing initial interpretations when coding references to "enjoyment and play" and "infant development" as parents sometimes described these concepts together, linking play to social development. Through discussion, the coders agreed to keep the codes distinct to better capture nuanced differences in how parents spoke about play as a component of development under the wider theme of infant outcomes, with infant development more closely linked to skill acquisition. CS and MR then came together to discuss initial articulation of themes. The analyses of both data sets were then brought together by NS and KRS and the themes further refined to offer further insights which led to the themes presented in this paper. For example, while codes such as “music therapy” remained in the final conceptualisation of subthemes, others were refined. For example, where the original analysis distinguished between codes such as “a music class”, “mixed music”, and “other”, looking at the data set as whole KRS and NS prioritised bringing out differences in the centrality of the musical aspects of the activities. This led to our description of the subthemes “Multi-practice activities that involve music” and “Music groups”.

## Results

### Survey respondents

#### Parents/Caregivers

578 respondents completed the parent/caregiver survey. As summarised in Table 2, geographically, the respondents were spread around the UK broadly in proportions that reflect those of the general population (as represented in the 2021 Census [64]) with a slight bias towards the smaller regions; 78% England, 8% Wales, 7% Scotland, 7% Northern Ireland (compared to 84% living in England, 5% in Wales, 8% living in Scotland, and 3% in Northern Ireland in the 2021 census). In terms of ethnicity, 82% reported being White (compared with 82% in the 2021 census). The majority (81%) identified as women and the mean age was 33.7 years (SD = 5.2) which aligns with the mean age for mothers who gave birth in England and Wales (30.90) according to the 2021 Census [65]. 69% of respondents had completed higher education, 6% (*n* = 36) considered themselves to have a disability as defined by the Equality Act 2010, and 32% reported having a household income of £20,000-£39,000. 86% reported being married or in a domestic partnership. In terms of general musical expertise, the average score on the Goldsmiths Musical Sophistication Index (Gold-MSI) was 71.0 which is within the standard deviation of 20.6 from the 81.9 mean score identified for the general population in Müllensiefen et al. ([66], p. 10). 16 participants (3%) reported working with music professionally.

**Table 2:** Sociodemographic characteristics of the parents/caregivers sample, *N* = 578

	***n* (%)**
*In which region(s) do you live? Please tick all that apply*	
**Scotland**	***40* (7%)**
Highlands and Islands	*5* (1%)
Northern Scotland	*12* (2%)
Southern Scotland	*23* (4%)
**England**	***451* (78%)**
North East England	*41* (7%)
North West England	*56* (10%)
Yorkshire and the Humber	*41* (7%)
East Midlands	*47* (8%)
West Midlands	*58* (10%)
East of England	*40* (7%)
South East England	*70* (12%)
South West England	*48* (8%)
London	*50* (9%)
**Wales**	***47*(8%)**
North Wales	*6* (1%)
Mid Wales	*3* (1%)
West Wales	*2* (0%)
South Wales	*36* (6%)
**Northern Ireland**	***40* (7%)**
**Would prefer not to say**	***2* (0%)**
*I identify myself as*	
Women	*467* (81%)
Men	*110* (19%)
Non-binary/transgender	*0* (0%)
Prefer not to say	*1* (0%)
*Relationship status*	
Single	*53* (9%)
Married or domestic partnership	*495* (86)
Widowed	*0* (0%)
Divorced	*3* (1%)
Separated	*10* (2%)
Other	*11* (2%)
Would rather not say	*6* (1%)
*Do you consider yourself to have a disability as defined by the Equality Act 2010?*	
Yes	*36* (6%)
No	*535* (93%)
Would rather not say	*7* (1%)
*I classify myself as…Please tick all that apply*	
**White**	***472* (82%)**
White—English/Welsh/Scottish/Northern Irish/British	*426* (73%)
White—Irish	*11* (2%)
White—Gypsy or Irish Traveller	*0* (0%)
Any other White Background	*35* (6%)
**Mixed/Multiple ethnic groups**	***24* (4%)**
Mixed/Multiple ethnic groups—White and Black Caribbean	*13* (2%)
Mixed/Multiple ethnic groups—White and Black African	*2* (0%)
Mixed/Multiple ethnic groups—White and Asian	*1* (0%)
Any other Mixed/Multiple ethnic background	*8* (1%)
**Asian**	***42* (7%)**
Asian/Asian British—Indian	*12* (2%)
Asian/Asian British—Pakistani	*14* (2%)
Asian/Asian British—Bangladeshi	*4* (1%)
Asian/Asian British—Chinese	*4* (1%)
Any other Asian background	*8* (1%)
**Black/African/Caribbean**	***38* (7%)**
Black/African/Caribbean/Black British—African	*25* (4%)
Black/African/Caribbean/Black British—Caribbean	*13* (2%)
Any other Black/African/Caribbean background	*0* (0%)
**Other ethnic group**	***5* (1%)**
Any other ethnic group	*1* (0%)
Arab	*4* (1%)
**Would rather not say**	***3* (1%)**
*Approximately, what is your yearly household income*	
Unemployed/Full-Time Student	*12* (2%)
Retired	*0* (0%)
Less than £10,000	*16* (3%)
£10,000-£19,000	*45* (8%)
£20,000-£29,000	*101* (17%)
£30,000-£39,000	*86* (15%)
£40,000-£49,000	*88* (15%)
£50,000-£59,000	*78* (13%)
£60,000-£69,000	*61* (11%)
More than £70,000	*65* (11%)
Would rather not say	*26* (5%)
*What is the highest educational and/or vocational qualification you have already attained?*	
Did not complete any school qualification	*1* (0%)
Completed first school qualification at about 16 years (e.g., GCSE)	*62* (11%)
Completed second qualification (e.g., A levels/BTEC/High School)	*116* (20%)
Undergraduate degree or professional qualification (e.g., bachelors degree/NVQ 6)	*275* (48%)
Postgraduate degree (e.g., masters, PHD, DMA, DMus degree, NVQ7)	*121* (21%)
I am still in education	*3* (1%)

#### Providers

50 respondents completed the provider survey. As summarised in Table 3, the majority were living in England (92%), with 4% in Wales, 4% in Northern Ireland and no participants from Scotland. 96% identified as women, 84% as White, and the mean age was 42.2 (SD = 9.09). Over half (56%) had been working in musical care for five or more years (with 36% doing so for 10 or more years). Most respondents (68%) had not had training in delivering musical care activities in the perinatal period or in early years and for just over half (54%), delivering musical care work was their primary source of income. The practitioners came from a variety of backgrounds in terms of training and experience (e.g., music therapist, play therapist, child development experts, parents, community musicians). 38% of respondents included “community” (most often as part of “community music”) in their description of their work and 14% of respondents included music therapy (Additional File 6). Given that “musical care” is a new umbrella term, there is no umbrella data that captures the combination of professionals targeted in this survey. However, in a study that mapped music therapists in the UK from 2017 (Carr et al., [67]), 93% were White, 78% identified as female, and 59% were living in England.

**Table 3:** Sociodemographic characteristics of the providers sample, N=50.

	*n (%)*
*In which region(s) do you live? Please tick all that apply*	
**Scotland**	*0* (0%)
Highlands and Islands	*0* (0%)
Northern Scotland	*0* (0%)
Southern Scotland	*0* (0%)
**England**	***48*(92%)**
North East England	*4* (8%)
North West England	*4* (8%)
Yorkshire and the Humber	*3* (6%)
East Midlands	*5* (10%)
West Midlands	*4* (8%)
East of England	*6* (12%)
South East England	*8 *(15%)
South West England	*3* (6%)
London	*11* (21%)
**Wales**	***2*** **(4%)**
North Wales	*1 *(2%)
Mid Wales	*0* (0%)
West Wales	*0* (0%)
South Wales	*1* (2%)
**Northern Ireland**	***2*** **(4%)**
**Would prefer not to say**	***0*** **(0%)**
*I identify myself as....*	
Female	*48* (96%)
Male	*2* (4%)
Non-binary	*0* (0%)
Other	*0* (0%)
Would rather not say	*0* (0%)
*I classify myself as…Please tick all that apply*	
**White**	***47*** **(94%)**
White - English/Welsh/Scottish/Northern Irish/British	*43* (84%)
White - Irish	*0* (0%)
White - Gypsy or Irish Traveller	*0* (0%)
Any other White Background	*4* (8%)
**Mixed/Multiple ethnic background**	***1*** **(2%)**
Mixed/Multiple ethnic groups - White and Black Caribbean	*0* (0%)
Mixed/Multiple ethnic groups - White and Black African	*0 *(0%)
Mixed/Multiple ethnic groups - White and Asian	*0* (0%)
Any other Mixed/Multiple ethnic background	*1* (2%)
**Asian**	*0* (0%)
Asian/Asian British - Indian	*0* (0%)
Asian/Asian British - Pakistani	*0* (0%)
Asian/Asian British - Bangladeshi	*0 *(0%)
Asian/Asian British - Chinese	*0* (0%)
Any other Asian background	*0* (0%)
**Black/African/Caribbean/Black British**	*2* (4%)
Black/African/Caribbean/Black British - African	*0* (0%)
Black/African/Caribbean/Black British - Caribbean	*0* (0%)
Any other Black/African/Caribbean background	*0* (0%)
**Any other ethnic group**	*0*** (0%)**
Any other ethnic group	*0* (0%)
Arab	*0* (0%)
**Would rather not say**	***1*** **(2%)**
*Approximately, what is your yearly household income*	
Unemployed/Full-Time Student	*0* (0%)
Retired	*0* (0%)
Less than £10,000	*3* (6%)
£10,000-£19,000	*3* (6%)
£20,000-£29,000	*7 (14%)*
£30,000-£39,000	*9* (18%)
£40,000-£49,000	*7* (14%)
£50,000-£59,000	*4* (8%)
£60,000-£69,000	*2 *(4%)
More than £70,000	*4* (8%)
Would rather not say	*11* (22%)
*What is the highest educational and/or vocational qualification you have already attained?*	
Did not complete any school qualification	*0* (0%)
Completed first school qualification at about 16 years (e.g., GCSE)	*2* (4%)
Completed second qualification (e.g., A levels/BTEC/High School)	*5* (10%)
Undergraduate degree or professional qualification (e.g., bachelors degree/NVQ 6)	*18* (36%)
Postgraduate degree (e.g., masters, PHD, DMA, DMus degree, NVQ7)	*24* (48%)
I am still in education	*1* (2%)
*Do you consider yourself to have a disability as defined by the Equality Act 2010?*	
Yes	*6* (12%)
No	*43* (86%)
Would rather not say	*1* (2%)
*How long have you been a musical care practitioner?*	
less than 6 months	*1* (2%)
6–12 months	*9* (18%)
1–5 years	*12* (24%)
5–10 years	*10* (20%)
10+ years	*18* (36%)
*Is delivering musical care your primary source of income?*	
Yes	*27* (54%)
No	*23* (46%)
*Do you have training in delivering musical care activities, in the perinatal period, or in early years work?*	
Yes	*24* (32%)
No	*52* (68%)

## Survey Responses

### Participation in and provision of musical care activities

#### Participation in musical care activities by parents/caregivers

549 (95%) parents/caregivers had heard of musical care activities offered during the beginning of life. Of these, most respondents (*n* = 475, 83%) had participated in formal musical care activities. Only a small number of activities listed [4] were attended by at least one third of respondents. Activities for babies up to the age of two years were most commonly participated in (*n* = 429, 90%), while just under half participated in activities for parent(s)/caregiver(s) expecting babies (*n* = 228, 48%). Combining the activities that take place in the postnatal period targeted at parents/cargivers and babies, 95% of the participants who had participated in any activity, did so in this period (*n* = 449).

As summarised in Table 4, activities that respondents participated in the most were those that included some music as opposed to those that focus only on music, such as *music sessions for babies*. In the postnatal period, *play and development groups that involve some music* were by far the most commonly attended activities (*n* = 332, 70% “for babies” and *n* = 235, 49% “for parents”). *Antenatal sessions that involve some music* were most commonly attended during the antenatal period (*n* = 143, 30%) but still substantially less than the play and development groups that occur during the postnatal period.

**Table 4:** Parent/caregivers’ participation in formal musical care activities

Formal musical care activities for	**n (%)**
…**babies **(*N *= 475)	
Play and development groups for babies that involve some music	*332* (70%)
Music sessions for babies	*195* (41%)
Baby massage that involves some music	*158* (33%)
Dance sessions for babies	*88* (19%)
Baby yoga that involves some music	*80* (17%)
Music therapy groups/individual sessions for babies	*29* (6%)
Music therapy in hospital for babies	*7* (1%)
Live music playing in hospital for babies	*5* (1%)
Other	*2* (0%)
…**parent(s)/caregiver(s) with babies** (*N *= 475)	
Play and development groups that involve some music for parent(s)/caregiver(s)	*235* (49%)
Music classes for parent(s)/caregiver(s)	*93* (20%)
Dance sessions for parent(s)/caregiver(s)	*45* (9%)
Choirs/singing groups for parent(s)/caregiver(s)	*28* (6%)
Music therapy groups/individual sessions for parent(s)/caregiver(s)	*22* (5%)
Music therapy in hospital for parent(s)/caregiver(s)	*9* (2%)
Other	*9* (2%)
Live music playing in hospital for parent(s)/caregiver(s)	*7* (1%)
Song writing/creative sessions for parent(s)/caregiver(s)	*6* (1%)
… **parent(s)/caregiver(s) expecting babies **(*N *= 475)	
Antenatal sessions that involve some music	*143* (30%)
Music classes/groups for expectant parent(s)/caregiver(s)	*67* (14%)
Music therapy groups/individual sessions for parent(s)/caregiver(s)	*33* (7%)
Dance sessions for expectant parent(s)/caregiver(s)	*26* (5%)
Music therapy in hospital for expectant parent(s)/caregiver(s)	*25* (5%)
Live music playing in hospital for expectant parent(s)/caregiver(s)	*24* (5%)
Choirs/singing groups for expectant parent(s)/caregiver(s)	*20* (4%)
Other	*18* (4%)
Song writing/creative sessions for expectant parent(s)/caregiver(s)	*6* (1%)

The most attended activities were also the ones that parents/caregivers most wanted more of. Out of those that wanted more musical care activities for each target group, most (40%) wanted more *Antenatal sessions that involve some music* while they were expecting a baby (*n* = 186), 42% wanted more *Play and development groups that involve some music for parents/caregivers* (*n* = 201), and 28% wanted *Play and development groups for babies that involve some music* (*n* = 138). More generally, 80% of parents/caregivers wanted more activities in the antenatal period and 87% wanted more for each of the target groups (parents/caregivers or babies) in the postnatal period.

Most parents/caregivers attended musical care activities with children 0–12 months (*n* = 341, 73%), weekly (*n* = 347, 74%). 14% (*n* = 65) only attended the activity once, 28% (*n* = 131) attended for 3–6 months, and 17% (*n* = 81) attended for 6 months to a year while 14% (*n* = 64) attended for over a year. About a quarter of the activities were free (27%, *n* = 124), 22% (*n* = 105) cost less than £5, and 44% (*n* = 204) cost £5–10. 7% (*n* = 34) cost £10 or more.

#### Provision of musical care activities

The most popular postnatal activities – *Play and development groups that involve some music* and *Music classes* – are also the most commonly offered by the providers. Strikingly, most of the providers do not offer antenatal activities. However, one of the most common antenatal activities offered *does* align with one of the most commonly attended antenatal activities (*Music classes/groups for expectant parent(s)/caregiver(s))* (Table 5).

**Table 5:** Providers’ formal musical care activities

Formal musical care activities for	***n* (%)**
…**babies** (*N* = *50)*	
Music sessions for babies	*33* (66%)
Play and development groups for babies that involve some music	*26* (52%)
Music therapy groups/individual sessions for babies	*10* (20%)
None	*8* (16%)
Baby massage that involves some music	*8* (16%)
Dance sessions for babies	*6* (12%)
Other	*5* (10%)
Baby yoga that involves some music	*4* (8%)
Live music playing in hospital for babies	*4* (8%)
Music therapy in hospital for babies	*2* (4%)
…**parent(s)/caregiver(s) with babies** (*N* = *50)*	
Music classes for parent(s)/caregiver(s)	*20* (40%)
Play and development groups that involve some music for parent(s)/caregiver(s)	*20* (40%)
Choirs/singing groups for parent(s)/caregiver(s)	*13* (26%)
None	*9* (18%)
Music therapy groups/individual sessions for parent(s)/caregiver(s)	*9* (18%)
Other	*7* (14%)
Dance sessions for parent(s)/caregiver(s)	*4* (8%)
Live music playing in hospital for parent(s)/caregiver(s)	*3* (6%)
Song writing/creative sessions for parent(s)/caregiver(s)	*3* (6%)
… **parent(s)/caregiver(s) expecting babies** (*N* = *50)*	
None	*31* (62%)
Choirs/singing groups for expectant parent(s)/caregiver(s)	*8* (16%)
Music classes/groups for expectant parent(s)/caregiver(s)	*7* (14%)
Antenatal sessions that involve some music	*2* (4%)
Live music playing in hospital for expectant parent(s)/caregiver(s)	*2* (4%)
Music therapy groups/individual sessions for expectant parent(s)/caregiver(s)	*2* (4%)
Music therapy in hospital for expectant parent(s)/caregiver(s)	*2* (4%)
Other	*2* (4%)
Dance sessions for expectant parent(s)/caregiver(s)	*1* (2%)
Song writing/creative sessions for expectant parent(s)/caregiver(s)	*1* (2%)

In open responses, providers gave many examples of the demographic characteristics of the people that usually participate in their activities. They described the majority as identifying as mothers, aged approximately 25–45, White, from families with employment unless the activities were targeted to particular groups such as people experiencing mental health challenges, children with additional needs, fathers, or in areas of social and economic deprivation. While most of the activities were attended by mothers, some were also attended by fathers and grandparents, with grandparents being often mentioned.

### Descriptions and experiences of musical care activities

In the description of the musical care activities, music could be either be part of a wider range of activities or it could be placed as the central activity. We identified four broad categories of musical care activities described by parent/caregivers and providers: multi-practice activities that involve music, music groups, live concerts primarily for babies, and music therapy (see Table 6 for a summary and Additional File 7: Codebook for examples). These reflect how music can either be seen as dominant in the activity or woven into a multi-practice session.

**Table 6:** Musical care activities

Music dominant and multi-practice activities
Multi-practice activities that involve music
Music groups
Live concerts primarily for babies
Music therapy

*Multi-practice activities that involve music* included baby yoga, baby development groups, or play groups. Within these classes that involve music, we see variation in how central or peripheral the music is. For example, one provider described:"I run a stay and play and for 20 min at the end we do themed songs depending what the theme is. We usually use different props or actions” (Provider, Entertainment and community, NW England, 32, Female). This example shows music more in the periphery as a process that closes the session. Another provider gave an example of music playing a more central role throughout a baby group:"Use song and rhymes to interact and develop the participation in the class. In Baby group use the same rhyme before starting so the babies become familiar with the activity such as baby massage, mother and baby yoga and Bath Babies” (Provider, Parent and baby classes, W Midlands, 39, Female).

The *music groups* included playing instruments, musical play, and singing. For example, one parent described that they attended “… a weekly class involving singing, exploring, finding rhythm, and making new friends” (Parent/caregiver, S.W. England, 37, Female). A music practitioner described their activity as “[m]usic & movement sessions which try & involve the parent/caregiver too. A mix of new & traditional songs & rhymes that encourage participants to dance/follow actions/play along with simple percussion instruments” (Provider, Informal music and movement classes, SE England, 57, Female). Within these types of musical care activities, music can be the exclusive focus or alongside other activities that share many musical characteristics (such as movement and dance).

Another prominent form of music-focussed activities was *live music concerts primarily for babies*. For example, a provider described running “Classical music in a relaxed atmosphere for babies, toddlers and young children. Concerts last 40 min and babies can crawl and explore whilst listening to the music” (Provider, Concerts for children and families, and music entertainment, SW England, 44, Female) and a parent described a “…concert at church – baby was very attentive to the music and the sounds"(Parent/caregiver, S.E. England, 50, Male). When described, the live music and presentational concert settings often included music from the Western Classical tradition.

There was very little mention of *music therapy* work by the parents/caregivers. Most of the descriptions of this work came from providers which catered to targeted groups. For example, one music therapist described a “Music therapy group for young homeless mothers and their babies…I have also run music therapy groups for mothers with postnatal depression and their babies, and community groups for mothers and babies” (Provider, Music Therapy, London, 47, Female).

### Motivations for, deterrents from, and perceived outcomes of musical care activity participation and provision

#### Motivations for and deterrents from musical care activity participation and provision

We identified four main themes related to motivators (“Personal preference”, “Experiential and practical factors”, “Recommendation by healthcare provider”, and “Expectation of benefit”) and two related to parents’ deterrents (“Resources and logistics”, and “Inclusivity and diversity”) from participation. We also identified four main themes related to motivations and challenges for musical care provision (“Personal experience”, “Professional experience”, “Gap in the market”, and “Coordination and collaboration”) (see Table 7 for a summary and Additional File 7: Codebook for examples).

**Table 7:** Personal and logistical factors of motivation and deterrence

**Parents’ motivators**		
Personal preference		
Experiential and practical factors		
Experience something new		
Get out of the house		
Convenient		
Recommendation by healthcare provider		
Expectation of benefit		
Perception that activity would be helpful		
Be part of a community		
Bonding		
**Parents’ deterrents**		
Resources and logistics		
Inclusivity and diversity		
**Providers’ motivators and challenges**		
Personal experience		
Professional experience		
Gap in the market		
Coordination and collaboration		

In terms of motivators, parents’/caregivers’ personal preference included liking music, either themselves and/or for their infant(s). Other motivators included factors associated with the practical experience of the activity itself (experiential and practical factors) such as wanting to experience something new (for the baby or the parents/caregivers), getting out of the house, and convenience. Parents also described being encouraged to participate by a healthcare provider/midwife. Other motivations were around the expectations of benefit. For example, music was seen as helpful for the baby, musical care activities were seen as being opportunities to meet other parents and being part of the community, or bond with their baby or their partner. Not all responses were positive, with deterrents including practical challenges of resources and logistics, and issues of inclusivity and diversity.

Practitioners described different reasons for running these groups primarily motivated by their personal experiences (such as having experience of being a parent or discussing their views on music) or professional experiences (such as being a music therapist). Some also described their motivation as addressing gaps in provision in their local area. For example, one parent provider described “Looking for classes to take part in with my 2 year old and finding a lack of classes in my area. I felt after lockdown children and parents really needed to socialise and get out of the house, so I started my classes” (Provider, Music and movement education, E of England, 37, Female). They also highlighted challenges in collaboration and coordination, such as issues of knowledge exchange and training.

#### Perceived outcomes of musical care activity participation and provision

Positive feelings of benefit reported in open responses included feeling creative, educated, excited, peaceful, proud, relieved, and satisfied. To understand parents’ and providers’ perceived social and emotional outcomes of engaging in musical care activities, we further asked a series of closed questions. Parents/caregivers agreed that, in broad terms, their positive and negative social and emotional states were positively affected by engagement in the musical care activity (Table 8). Respondents reported the greatest change in feelings of closeness with their baby and their own happiness.

**Table 8:** Perceived effect of chosen musical care activity

*Please indicate below whether or not participation in the musical care activity affected you in these ways*	*mean*	*SD*	*n*
Close to my baby	1.89	0.89	*464*
Happy	1.95	0.78	*466*
Connected to other people	2.31	0.98	*458*
Relaxed	2.32	0.98	*462*
Confident	2.41	0.86	*451*
Close to my partner (if applicable)	2.55	0.91	*193*
Anxious*	3.70	1.21	*392*
Worried*	4.09	0.96	*388*
Lonely*	4.23	0.9	*391*
Depressed*	4.32	0.89	*326*

1 = felt much more, 5 = felt much less; * negative statements

In terms of outcomes, through responding to closed questions, parents/caregivers agreed most strongly that by participating in a musical care activity they had done something for their baby/child and done something new (Table 9). Other positive outcomes described in the following open question included respondents feeling that they had done something for their partner and visited a new place.

**Table 9:** Perceived outcomes after taking part in chosen musical care activity (*N* = 467)

*Please indicate below whether or not you agree/disagree with the following statements about your participation in the musical care activity:*	*mean*	*SD*
I did something for my baby/child	1.40	0.63
I did something new	1.62	0.69
I got to know and understand my baby better	2.19	0.89
I did something for myself	2.28	1.10
I learnt new music	2.34	1.06
I saw a different side to my baby	2.37	0.94
I met new friends	2.61	1.17
*I didn’t get anything out of it	4.14	0.89
*I didn’t like it	4.18	0.93

1 = strongly agree, 5 = strongly disagree

^*^ negative statements

The perception of positive outcomes of music engagement seen in the motivations for attending activities were also seen in the descriptions of the outcomes of participation. In response to an open question, parents and providers described three kinds of positive outcomes: parent outcomes, infant outcomes, and parent-infant bonding. Parents also described negative experiences (see Table 10 for a summary and Additional File 7: Codebook for examples).

**Table 10:** Seeing individual and social experiences and outcomes

**Percieved outcomes**
**Parent/caregiver Outcomes**
Learning and gaining confidence
Building a community
Enjoyment
Relaxation
Support mental health
**Infant Outcomes**
Enjoyment and play
Engagement and socialising
Relaxation
Infant development
Music development
For baby in utero
**Parent/caregiver-Infant bonding**
**Negative experiences**

In terms of positive outcomes, parents reported learning about themselves and about how to make music and play with their babies. They also reported gaining confidence, building a community, experiencing enjoyment and relaxation, and addressing mental health challenges. Impact on mental health outcomes included the perspectives of partners, such as: “When my wife was pregnant we went to a music therapy type group for expectant mums, purely as my wife was stressed a lot, the music was the reason that all mums went, and to be fair it was good, but I think the mums and dads enjoyed comparing stories and stresses, so overall was a really handy thing” (Parent/caregiver, East of England, 36, Male). One provider described an “[i]ndividual music therapy session with a mum and her newborn—mum suffering from severe PPD [postpartum depression] and psychosis and in a catatonic state. Helped mum to acknowledge baby by singing to him—Mum shared she used to go church so I sang a hymn to which she spontaneously joined in” (Provider, Music therapy, London, 38, Female).

Infant outcomes included their enjoyment and play, engagement and socialising, relaxation, infant development, music development (including exposure to music), as well as being for the baby in utero. As an example of infant development, one provider described the aim of their work as being"…to provide a class that is more than just singing nursery rhymes. Giving children the chance to experience a real live instrument and help their development using musical techniques" (Provider, Music education, Yorkshire and the Humber, 35, Female). As an example of outcomes for babies in utero, one parent described work that began in hospital and traced the role of musical care activities that followed. “When I was pregnant with my little girl, her movements had stopped/slowed down. I had to attend hospital to be monitored. After half an hour, she had moved twice. As soon as they started to play music in my room, she kept kicking and turning. I was so relieved. […] I was told my baby liked music as she moved loads when it happened. So was told to try and play music as much as I can” (Parent/caregiver, Yorkshire and the Humber, 33, Female).

Parents and providers discussed positive impacts on the support of parent-infant bonding on themselves, and on their infant. For example, one parent described that “[i]t was lovely to spend time bonding with my child…” (Parent/caregiver, West Midlands, 31, Female). This aligned with the aims described by the providers, such as for “…parents to"tune in"to their babies" (Provider, Community-based music, E Midlands, 55, Female).

Providers' description of the aim of their work usually included a mix of several of the outcomes. For example, one provider described that their activity “…was aimed at those parents to enjoy and relax by being silly without feeling embarrassment, which was harmless and good fun. It was vital for those deaf children to watch their parents or grandparents act, which is perfectly normal. It is the same way for those hearing babies whose parents have been using their silly voices to make hearing babies laugh or giggle. At the end of the session, I add the music linked to the nursery rhymes which is suitable for those babies and parents to follow. At the end of the day, those children (deaf and hearing) are learning the language's developments at same time which is so vital” (Provider, BSL tutor teaching nursery rhymes with music, Northern Ireland, 55, Female).

Several parents/caregivers commented on aspects that were more challenging when participating in musical care activities. These included that parents/caregivers or infants did not enjoy the experience (because they found it stressful, they felt self-conscious, they were bored, or that they did not like the music). Parents/caregivers described in an open question increased negative feelings including feeling annoyed, irritated, awkward, and uncomfortable, and some were connected to personal preference (e.g.,"It was too 'airy fairy' for me! I prefer lively music. My son was very confused by it!" (Parent/Caregiver, S Wales, 41, Female)). Additionally, parents commented that, while their baby seemed to enjoy the activities, they did not. For example, one commented that “I remember feeling a bit embarrassed, but my baby loved it which is the main thing …It was out of my comfort zone and I felt embarrassed” (Parent/caregiver, SW England, 28, Female). Another parent commented that “I got joy from seeing her so happy and involved but really these sessions are boring for parents” (Parent/caregiver, Northern Ireland, 36, Female).

## Discussion

A wide range of activities during the beginning of life that include music currently happen in the UK, and the reasons that parents/caregivers choose to go range from those specific to music and its care potential to it being a leisure activity, often with a focus on the infants rather than their parents.

### Participation and provision

Most of the respondents had participated in at least one musical care activity during the beginning of life, and most that had heard of an activity had also participated in it. Though a wide range of activities was described, a relatively small number of the activities listed were attended by a substantial proportion of respondents. Most respondents attended activities in the postnatal period with far fewer doing so during pregnancy despite the documented importance of support at this stage [68]. Additionally, more respondents attended activities described as for their babies than for themselves. This pattern mirrors how outcomes of musical care activities are often divided in research and may reflect documented challenges of professional collaboration between midwifery, health visiting, nursing, and paediatrics [69]. This seperation between infant and parent benefit seem not to be reflective of how parents/caregivers describe their experience with some respondents beginning their descriptions with talking about doing an activity for their baby and then reporting on effect on both parents/caregivers and babies. Despite the emphasis on infant experiences in their descriptions of the musical care activities, parents/caregivers reported that they wanted more activities focussed on them as parents (see also [70]).

In contrast to the participants typically represented in the research literature, grandparents and fathers were mentioned as attendees at these musical care activities. Most parents/caregivers attended the musical care activity they discussed weekly, for three to six months reflecting the intensity and time-sensitive nature of this experience.

Most providers had not had training in musical care during the beginning of life, a pattern also observed in other socially engaged arts work [71]. This may reflect that much of this work is community led and a personal response to seeing an opportunity to provide community activities. It may also reflect the situation that, though there are formal music therapy and other programmes provided by charitable organisations (e.g., Spitalfields Music), these can come with high costs, can have specific entrance criteria, and have typically had limited geographic reach. It is similarly striking that only just over half of the providers had musical care delivery as their primary source of income, suggesting that for many this work was part of a portfolio career and for almost half, it was not the dominant part of that portfolio. In light of the skills required to facilitate musical care activities at the beginning of life, particularly for families who may be experiencing challenges, there is a clear need for additional practitioner support and training opportunities for those working outside of formal music therapy professions [72].

### Descriptions and experiences

Four broad categories of musical care activities were identified: Multi-practice activities that involve music, music groups, live concerts primarily for babies, and music therapy. The descriptions of the musical care activities include multiple types of engagement (singing, playing, stretching, sign language). Music could be placed as the central activity or part of a wider range of activities. Indeed, respondents named activities without including “music” but when they described what happened in the sessions, music seemed to have a central place. Other activities described as “music” also included other nearby activities (such as movement/dance). It is possible that these descriptions are connected with how activities are advertised and funded, or what gap they are addressing. The most commonly attended activities both pre- and postnatally were those that involved, rather than focussed on, music. Relatively few parents/caregivers discussed music therapy activities, but they were discussed by the providers. This may reflect the likelihood of accessing this targeted musical care practice that is often intended for a subgroup of participants (for example, children experiencing developmental delay and their parents [73]).

### Motivations for, deterrents from, and expectations of outcomes of musical care activities

Parents described personal preference, experiential and practical factors, recommendation by healthcare providers, and expectation of benefit as motivators for attending musical care activities. These aligned with the aims of the activities described by the providers. While some factors were seen as motivators, those same or very similar factors could be experienced as deterrents. For example, musical care activities were seen as an opportunity for socialising for those that felt part of a community while others felt that groups were not inclusive. Indeed, there is increasing awareness and development of music activities tailored to cultural and linguistic needs [74]. Other deterrents for parents included issues of logistics and resources, aligning with some of the resource challenges faced by providers. In addition, providers discussed challenges associated with collaboration and coordination among organisations, stakeholders, and areas of expertise, including issues of training. The exploration of the barriers and opportunities in participating and providing musical care requires more space than available in this article and are therefore explored in more detail in Sanfilippo, Spiro et al., Barriers and opportunities to accessing and providing musical care during the beginning of life: A mixed-methods survey study (in preparation).

The closed questions, which were developed in relation to existing literature that had at least begun to explore these areas,^1^ suggested overall positive outcomes for parents/caregivers, infants, and the relationship between them. Several areas discussed in the open responses have also had some interest from researchers and in some cases, have had substantial research investigating how these kinds of interventions can impact participants, either positively or negatively. For example, Parent/caregiver outcomes (Learning and gaining confidence [75], Building a community [76], Enjoyment [76], Relaxation [77], Support mental health [36, 39]); Infant Outcomes (Enjoyment and play [38, 39], Engagement and socialising [78], Relaxation [79], Infant development [48], Music development [80], Interest in babies in utero has been in the context of what might be perceivable [25]); Parent/caregiver-Infant bonding [35].

The open questions allowed for more discussion of negative experiences than often seen in the literature [81], suggesting that there is a more variegated range of experiences than usually represented. The more negative experiences were at least in part associated with preference, highlighting that musical preference is an important driver in choosing to attend these activities in general and in how they are experienced in particular [82]. Infant and caregiver enjoyment in particular was mentioned both positively and negatively. Indeed, though there is mounting evidence supporting the possible roles of musical care during the beginning of life, one cannot assume that everyone will benefit from a musical care activity or, even if they do, that they would benefit in the ways previously reported in the literature [83].

### Musical care and stepped care

This is the first research study that uses the term ‘musical care’ as a conceptual framework and as part of a survey. The responses to the survey suggest that parents/caregivers and providers understood the term, indicating that it is useful for this context. The co-constructed list of musical care activities along with the activities described in the open responses to the surveys suggests a rich and variegated landscape of musical care at this life stage. The practices range from targeted work that can happen in formal medical settings, to community provision either through large or small charitable organisations or individual freelancers. Musical care activities during the beginning of life blur the boundaries of work between this work as a health intervention, an artistic musical experience, and a diverting leisure experience. Indeed, the reasons that parents/caregivers choose to go to these activities range from those specific to music and its care potential to it being a leisure activity. The practices range from work that focusses on music, to multi-modal work and can be tailored to different populations and their needs. This landscape of work may then lend itself to a stepped-care approach [84] that allows families to access provision in a way that suits their needs. In a stepped care approach, more specialised professionals such as music therapists would provide more intensive/specialist services while community musicians provide lower intensity care. Individuals can move up and down the steps in relation to their needs. Furthermore, this range of work lends itself to supporting people who otherwise might remain unengaged, such as those who do not wish to disclose mental health challenges [15], have a distrust of formal mental health provision [85], or those who are struggling but do not feel they require formal intervention. This type of stepped care approach would need significant policy support and is discussed in co-developed policy recommendations that build on this project [86].

### Limitations and future work

The sociodemographic characteristics of the respondents to the parents/caregivers survey broadly aligns with key characteristics of the UK population. Though we emphasised the regions with smaller populations, the result is that we have rather different numbers in different categories. For example, most of respondents identify as “White”. Furthermore, we shared the surveys online in English. This method brings its own limitations of reach (in terms of language proficiency or having access to an online form). We also primarily shared the parents/caregivers survey through Prolific which has a bank of responders, a method which also has its own limitations [87]. Though we had respondents from a wide socioeconomic range, participation in research takes time which could have been a barrier in itself.

The respondents to the providers survey were from a more limited sociodemographic background, with most being from England, and identifying as White and female. Though more dominated by female and White respondents than sociodemographic data of the music therapy sector, the demographic characteristics of the respondents follow similar trends to that of the sector [67]. We aimed for a wide reach and therefore used surveys. Future research could use surveys – which have their own limitations [88] – alongside other methods, such as focus groups, to ensure more inclusive understanding of perspectives. It could also use methods of recruitment that engage participants and organisations from a range of backgrounds in a more tailored way. It could take an ecological approach to mapping creative practices that includes surveys, interviews, and consultations ([89], p. 1, see also [90]), and also use publicly available data about music provision, funding, and needs to identify under-resourced areas [91].

This article does not include analysis of everyday forms of musical care such as those incorporated into daily childcare and family routines (e.g., everyday music listening or singing at home). Such informal musical care has been investigated elsewhere [92–94] and future work could further explore the extent of fluidity between and potential mutual impact of formal and informal musical care practices during the beginning of life. This survey was shared with a general population. Therefore, some targeted practices – especially the vast range of specialist music therapy work discussed in the literature, such as infant-directed singing modelling [41–43] and music therapy in the neonatal intensive care unit (e.g., 47) – are not fully represented in this sample. This focus on the general population likely also contributed to the fact that this approach did not capture the full range of possibilities of reasons for accessing musical care activities or the range of pathways of referral. Future work could focus on the subgroup of people who have been referred to musical care activities. This would bring with it possibilities of exploring the range of mental and physical health profiles, their needs and expectations, which types of musical care provision they are referred to and have access to, and their experiences.

Despite these limitations, these findings point to some clear next steps. Though there has been much previous research on some musical care activities during the beginning of life, particularly music listening and music therapy, others have been less researched, including multi-practice activities such as play and development groups which involve some music. With many parents/caregivers participating in these types of musical care activities, research on these multi-practice activities is urgently needed. In terms of practitioners, the findings suggest that many are working in this area as part of a portfolio career. Research is needed to understand how to best support providers in this area in general and to identify and support their training and development needs. Finally, while examples of stepped-care approaches are being developed in music based organisations [95] and can be seen in current mental health provision [96], much more research is needed to understand how to implement and scale musical care nationwide. Through a long-term and coordinated response nationally and locally, implementation and scale would need to attend to local needs and be done in a way that connects with other pathways to improving support for children, parents, and families during this period [2].

## Conclusions

There is a broad range of musical care activities available during the beginning of life in the UK. Reasons for attending range from those specific to music and its care potential to seeing them as leisure activities. The findings have implications for the flexibility and role that musical care activities can play during the beginning of life and call for investigation into how they may be integrated into care. A stepped care approach could support more people to access them in ways that suit their needs. Developing a stepped care approach would require sufficient funding, collaboration, training, and support to ensure the variety of musical care activities required across all steps are equitably accessible and sustainable, with continued investment in research for an evidence-based approach.

## Authors'information

NS: Researcher in music and health and parent.

KRS: Researcher in music and health and healthcare innovation.

CS: Researcher in music and health.

MR: Researcher in music and health.

EC: Music therapy practitioner and researcher.

RP: Researcher in music and health and parent.

ET: Founder of App for parents and a parent.

## Supplementary Information

Additional File 1

Additional File 2

Additional File 3

Additional File 4

Additional File 5

Additional File 6

Additional File 7

## Data Availability

The data files for this project are available on RCM Online (10.24379/RCM.00002606) with some data redacted to protect respondents’ anonymity.

## Footnotes

1 For examples for each response category see: Anxiety and worry [97, 98], Closeness to baby [56, 99], Closeness to partner [100], Confidence [75], Connection to other people [39], Depression [36, 39], Happiness, Loneliness [39], Relaxation [77].
