# Supplementary material for: The landscape of musical care during the beginning of life in the United Kingdom: a mixed-methods survey study
Source: BMC Complement Med Ther. 2025 Oct 16;25:380. doi: 10.1186/s12906-025-05014-6 (PMC12532952; doi:10.1186/s12906-025-05014-6)
Supplement: Supplementary file 2 — Additional File 2 [file 12906_2025_5014_MOESM2_ESM.pdf]

Spiro, N.,* Sanfilippo, K.R.M.,* Shaughnessy C., Rowles, M., Coombes E., Perkins, R., & Tredget, E., (2025) The landscape of musical care during the beginning of life in the United Kingdom: A mixed-methods survey study, *BMC Complementary Medicine and Therapies*. doi: 10.1186/s12906-025-05014-6

Additional File 2: Consultation
Indicative focus group prompts

Today we are consulting with you to help develop the two surveys.

1. We would like to open the survey with a question that collates the different kinds of musical care activities that people have heard of. We're intentionally broad and we'll give them our definition.

"By musical care practices, we mean music listening as well as music-making activities that support any aspect of people's developmental or health needs: for example, physical and mental health, cognitive and behavioural development, and interpersonal relationships. In this work beginning of life refers to pregnancy through the early years of life."

Draft wording: "What musical care activities for families expecting babies and parents of young babies have you heard about?"

a. What do you think of the phrasing of the question?
b. A how should we invite responses, (e.g., lists, restrictive/comfortable, open text box etc.)

2. Second survey question could be an experience question. The goal of this question is to provide space for some detailed narratives of peoples' experiences.

Draft wording: "We'd like you to think about a specific musical care activity that you've engaged in. Focusing on that experience, could you describe the activity and tell us about **what it was like for you/how it affected you, if at all**?"

a. What do you think of the phrasing of the question?

3. Recruitment:

a. How would you suggest we share this survey to reach the widest range of participants (practitioners, minority/seldom heard voices).
b. Can you help with this step?

4. We would like to collect data about what practitioners do.

Draft wording: Please describe one musical care activity that you run and for what aim(s)?
a. What do you think of the phrasing of the question?

5. What other information should we collect
    1. Advertising activities (outcome vs. experience, fun vs. "goal" oriented).
    2. Quality (e.g., worked / not. How likely are you to recommend it? How would you know that it's working?, Frequency and regularity: Did you try as one-off or continued with?
    3. Access questions (How come across/access, was it free/not)
    4. Extra materials (e.g., Do you know of any writing/evaluation about this activity, is there a website?)

    5. Description of activity (e.g., leader experience, focus (on infants/parents/both), cultural awareness,)

6. How should we phrase a question about the role of evidence in choosing to participate, advertising activities, setting up and growing (implementation and upscaling) work.
    1. Rating scale: how important is it to you that the activity has external evaluation/friend recommended etc.
    2. How important is it to you that musical care in general has research evidence?
    3. How important is it to you that musical care in specific area (parental mental health) has research evidence?
    4. How important is are different evaluator types? (e.g., participants, externals, formal evaluation processes)]
    5. Open box
    6. Practitioner vs. participant directed questions.

7. We will ask a question about implementing and sustaining musical care work. We propose this wording: "How can we implement more musical care activities, sustain and upscale them in the future?"

a. What do you think of the phrasing of the question?

b. How should we invite responses, (e.g., lists, restrictive/comfortable, open text box etc.)

8. How do you think we should ask about barriers and enablers.

9. What would you like to know about barriers to providing or accessing musical care activities?
    1. Awareness of musical care activities
    2. Practitioners' experience being explicit (e.g., number of years working)
    3. Practitioners' area of training being made explicit (e.g., music therapy)
    4. Individual's confidence to participate in music
    5. Clarity of skill level needed to participate in the activity
    6. Accessibility (e.g., wheelchair accessible)
    7. Cost to participant
    8. Funding for musical care activity
    9. Language
    10. Culture
    11. What would help to overcome barriers?
    12. What are the enablers to providing or accessing musical care activities?

10. Is there anything else you'd like to add?
