# Supplementary material for: The landscape of musical care during the beginning of life in the United Kingdom: a mixed-methods survey study
Source: BMC Complement Med Ther. 2025 Oct 16;25:380. doi: 10.1186/s12906-025-05014-6 (PMC12532952; doi:10.1186/s12906-025-05014-6)
Supplement: Supplementary file 3 — Additional File 3 [file 12906_2025_5014_MOESM3_ESM.pdf]

Spiro, N.,* Sanfilippo, K.R.M.,* Shaughnessy C., Rowles, M., Coombes E., Perkins, R., & Tredget, E., (2025) The landscape of musical care during the beginning of life in the United Kingdom: A mixed-methods survey study, *BMC Complementary Medicine and Therapies*. doi: 10.1186/s12906-025-05014-6

Additional File 3: Consultation

Musical care at the beginning of life:
Mapping and upscaling resources
Feedback form

1. Invitation and Consent
2. Perspective
3. Musical care activities
4. Dissemination
5. Experiences of musical care activities
6. Barriers and opportunities
7. Demographics
8. Sources of support.

1. Invitation and Consent

Invitation

You are being invited to take part in my research project. Before you decide, it is important for you to understand why the research is being done and what it will involve. Please take time to read the following information carefully and discuss it with others if you wish. Ask us if there is anything that is not clear or if you would like more information. Take time to decide whether or not you wish to take part. Thank you for reading this.

Project

The project looks at musical care at the beginning of life. Musical care refers to the role of music—music listening as well as music-making—in supporting any aspect of people's developmental or health needs: for example, physical and mental health, cognitive and behavioural development, and interpersonal relationships. We are interested in musical care activities during pregnancy as well as with parents and caregivers and their infants. In the surveys we plan to ask about musical care during the antenatal period up to 2 years, with the surveys going to anyone with a youngest child aged up to 5 years.

The starting point of the project is that availability of musical care at the beginning of life (e.g., in mum and baby groups, music therapy or other activities) is not equally distributed around the UK, that activities are not always clearly visible and that there are barriers to participation. This project focuses on addressing these gaps by exploring how musical care in the beginning of life can be implemented and upscaled. This is a three-part project involving a consultation phase, a mapping exercise (through online surveys) and a knowledge exchange step. Here we are inviting you to the first part. You will be invited to subsequent parts as well but participation in the consultation phase does not mean you have to continue participating in later parts.

Nature of participation

This project will last one year. But at this stage we are only asking you to complete a feedback form which we envisage should take about 30-45 minutes. The feedback form includes consideration the following areas: (1) collation of existing musical care provision, (2) recruitment, (3) experience of existing musical care provision (4) information about musical care provision, and (5) opportunities for rolling out musical care during the beginning of life. Key ideas from the feedback forms will be used anonymously to inform the development of the online surveys that will be used in the mapping exercise. Participants who complete participation in this consultation step will be offered a £20 online voucher

The subsequent surveys will be launched as soon as possible after the consultation phase and the knowledge exchange events are planned for spring and summer 2022.

Characteristics of participants

We are contacting you because we are looking to bring together a range of experts in musical care at the beginning of life including parents/carers, experts in public health policy, experts in health care implementation, and musical care providers (music therapists, music in health practitioners, etc.).

Voluntary participation

It is up to you to decide if you want to take part in this project or not. If you don't want to take part, or you change your mind about taking part, having agreed to do so, you won't be penalized in any way. If you do decide to take part, you will be given this information sheet to keep and be asked to sign a consent form. If you decide to take part you are still free to withdraw at any time, without giving any reason. You can withdraw either by stopping your completion of the feedback form and/or by withdrawing consent for me to use whatever contribution you have already made to the research. Again, you won't be penalized in any way.

Lifestyle restrictions

We do not envisage that participation in this study will lead to lifestyle restrictions.

Potential risks to participants

We do not envisage any risks from taking part in this project, but completing the form might lead to reflections about sensitive aspects of topics such as your experience of early parenthood. If you feel uncomfortable and would like to stop filling in the form, you can do so at any time. In the event that participation in the research causes any emotional distress, you can seek emotional support from the following sources:

- Your GP, midwife or health visitor
- Accident and Emergency at the nearest hospital or call 999
- The Samaritans: 116 123 or email jo@samaritans.org. They are open 24 hours a day, 365 days a year, to listen to anything that is upsetting you for free and won't appear on your phone bill).
- PANDAS: 0808 196 1776 (11am-10pm, a source of support specifically for post-natal mental health).
- The charity Mind's pages on postnatal depression and perinatal mental health: https://www.mind.org.uk/information-support/types-of-mental-health-problems/postnatal-depression-and-perinatal-mental-health/about-maternal-mental-health-problems/
- The NHS pages on post-natal depression: https://www.nhs.uk/conditions/post-natal-depression/

Potential benefits to participants

While people taking part in our project are unlikely to experience any personal benefits as a result, we hope our research will inform development and upscaling of musical care at the beginning of life in the UK.

Possible termination of research

If the project has to be terminated for any reason you will be informed and told why. Any information provided by you until the point of termination could still be used for the purposes of the research.

Confidentiality and anonymity

Information collected about you during this consultation phase, will be kept strictly confidential. Information you provide will only be attributed to you by name with your explicit permission. The only time that confidentiality would be broken is in the event that you disclose risk of immediate harm to yourself or others in which case we may need to discuss this with somebody else.

Storing personal data and information

Any information that you provide for the purposes of the research will be stored securely on password protected cloud storage at the Royal College of Music (RCM) for 10 years. If we wish to re-use it within this time period, we will seek your permission to do so. At the end of the period, it will be destroyed.

Outputs

The results of the project will be published in academic journals and books as well as in other forms (i.e., reports, presentations, websites) in the public domain. You will not be identified by name or other identifying feature in any publication. If you are interested, we can send these results to you once the project is complete.

Ethical approval

The Conservatoires UK Research Ethics Committee (CUK REC) has reviewed this project and granted ethical approval for it to be carried out.

For further information on the study

Contact:

Neta Spiro or Rosie Perkins
Centre for Performance Science, Royal College of Music, Prince Consort Road, London, SW7 2BS
Emails: neta.spiro@rcm.ac.uk
To speak to someone outside the immediate study team or to raise a concern, please contact:
Prof Aaron Williamon, Head of the Centre for Performance Science, Royal College of Music, Prince Consort Road, London, SW7 2BS Email: aaron.williamon@rcm.ac.uk

Thank you for reading this Participant Information Sheet and for considering your participation in this research project.

- ☐ I confirm that I have read and understood the participant information sheet for this research project and know I can contact the researchers if I have questions
- ☐ I understand that my participation is voluntary and that I am free to withdraw at any time without giving any reason.
- ☐ I give the researcher(s) permission to collect information about me and from me for the purposes of the research project provided all information about me will be kept confidential, stored securely and destroyed after 10 years.
- ☐ I agree to take part in the above-named project.
- ☐ Name of participant
- ☐ Signature
- ☐ Date

We’re developing two online surveys to find out about musical care activities for families expecting babies and parents of young babies. One is to learn from families and the other from practitioners. Through the surveys we would like to map people’s experiences of musical care activities in this life stage. The results of the surveys will lead to the development of recommendations for implementing and upscaling these musical care practices across the UK.

By musical care practices, we mean music listening as well as music-making activities that support any aspect of people’s developmental or health needs: for example, physical and mental health, cognitive and behavioural development, and interpersonal relationships. In this work beginning of life refers to pregnancy through the early years of life. We are interested in musical care activities during pregnancy as well as with parents and caregivers and their infants. In the surveys we plan to ask about musical care during the antenatal period up to 2 years, with the surveys going to anyone with a youngest child aged up to 5 years.

Here we are consulting with you to help develop the two surveys. We’re interested in what topics we should ask about, specific questions we should include, who we should send these surveys to and how to reach a broad demographic range. We are hoping for your detailed input and so we expect this to take around 30-45 minutes. You can leave the form and pick up where you left off if you are signed into a Google account.

2. Perspective

1. Are you joining this because you are (please tick all that apply):
   - ☐ Parent?
   - ☐ Professional that works in the area of musical care during the beginning of life?

2. If you are a professional that works in the area of musical care during the beginning of life, please tell us what you do (e.g. music therapist, perinatal psychologist)

3. Musical care activities

We want to open the surveys with a question that collates the different kinds of musical care activities that people have heard of. We're intentionally broad and we'll give them the following definition. "By musical care practices, we mean music listening as well as music-making activities that support any aspect of people's developmental or health needs: for example, physical and mental health, cognitive and behavioural development, and interpersonal relationships. In this work beginning of life refers to pregnancy through the early years of life."

Proposed question. "What musical care activities for families expecting babies and parents/caregivers of infants have you heard about?"

3. a. Should we change how the question is worded and if so, what would you propose instead?
   - ☐ No, it's fine as it is
   - ☐ Yes (please suggest new wording in the box below)

   b. Please suggest new wording here:

4. How should we invite responses to this question?

☐ List

☐ Open box

5. If the consensus is that a list would be best, this is our proposed list. Where appropriate, these could be for either babies or parents/caregivers or families:
   - Antenatal sessions
   - Antenatal music sessions
   - Baby yoga, baby massage
   - Choirs/singing groups
   - Dance sessions
   - Informally singing or music making with baby/child
   - Listening to music (on a personal device/at home)
   - Listening to music at a formal event, e.g., a concert
   - Live music playing in hospital
   - Music classes
   - Music therapy groups/individual sessions
   - Music therapy in hospital
   - Song writing

6. Are there musical care activities included in the list that you would exclude? If yes, please list them here and talk a bit about why they should be excluded. For this question, and for all other open questions, please write in as much detail as possible.

7. Are we missing any musical care activities?
   If yes, please list them here and talk a bit about why they should be included.

8. Are there musical care activities that you would describe differently?
   If yes, please list them here, including both our description and your suggestion.

4. Dissemination

9. How would you suggest we share these surveys to reach the widest range of participants (practitioners, minority/seldom heard parents)?
   For example, which organisations should we contact, is there any particular aspect of the project you recommend we highlight etc. (Please write in as much detail as possible.)

10. Can you help us share this survey? e.g., send the link to your friends, professional networks, feature this survey in a newsletter.
    - ☐ Yes
    - ☐ No

5. Experiences of musical care activities

Another key question in the survey for parents/caregivers will be about their experience of a particular musical care activity. The goal of this question is to hear some detailed narratives of peoples' experiences. We've come up with this phrasing: "We'd like you to think about a specific activity that you've engaged in. Focusing on that experience, could you describe the activity and tell us about what it was like for you/how it affected you, if at all?"

11. Do you have a preference between "what it was like for you" and "how it affected you, if at all"?
    - ☐ I prefer "what was it like for you"
    - ☐ I prefer "how it affected you, if at all"
    - ☐ Would an alternative phrasing be preferable and if so, what would you propose? Please write your alternative wording here:

After the open box, we'll have a list of possible outcomes where people can indicate whether or not and how participation in the activity affected them. We will ask about change (more, less, no change) and about their agreement with the
statements.
"I felt more/less/just as..."

- anxious
- close to my baby
- close to my partner
- depressed
- happy
- lonely
- relaxed
- socially connected
- worried

"I agree/disagree that"

- I learnt new music
- I did something for myself
- I did something for my baby/child
- I did something new
- I didn't like it
- I didn't get anything out of it
- I met new friends

12. Which additional outcomes would you recommend?

13. What additional information would you want to collect about the musical care activity described in the experience question above. For each of these please rate how helpful or unhelpful this information would be to collect. 1 = Unhelpful, 5 = Helpful

14. Participants' recommendation of the activity to others (e.g. Ask participants to rate if they would strongly recommend to strongly not recommend using a rating scale)

Unhelpful = 1	2	3	4	Helpful = 5

15. Frequency and regularity (e.g., for parents: Did you try as one-off or continued with?; How many sessions did you attend?)

Unhelpful = 1	2	3	4	Helpful = 5

16. How was it advertised (for practitioners)/How did you find out about it (for families)

Unhelpful = 1	2	3	4	Helpful = 5

17. Cost (e.g. was it free, not free)

Unhelpful = 1	2	3	4	Helpful = 5

18. Leader experience (credentials)

Unhelpful = 1	2	3	4	Helpful = 5

19. How long the activity has been running

Unhelpful = 1	2	3	4	Helpful = 5

20. Focus of the activity (on infants/parents/both)

Unhelpful = 1	2	3	4	Helpful = 5

21. Cultural inclusivity (e.g., How your culture has been taken into account (families)/ How the culture of the local community has been taken into account (practitioners))

Unhelpful = 1	2	3	4	Helpful = 5

22. If an evaluation has been done

Unhelpful = 1	2	3	4	Helpful = 5

23. Form of evaluation (e.g., Personal accounts, Internal evaluation, External formal evaluation)

Unhelpful = 1	2	3	4	Helpful = 5

24. Extra materials (e.g., Do you know of any writing/evaluation about this activity; Is there a website?)

Unhelpful = 1	2	3	4	Helpful = 5

25. Any other information we should ask about or further comments:

6. Implementation and barriers

26. We will ask a question about implementing, sustaining and scaling-up musical care work. We propose this wording: “How can we implement more musical care activities, and sustain them in the future?”
    Should we change how the question is worded and if so, what would you propose instead?

27. We will also ask the question “What are the barriers to providing/accessing musical care activities?” Please tell us what areas we should ask about. Please list as many as you think are appropriate - the text box will expand.

28. Is there anything else we should include in these surveys?

7. Demographics

29. In which region do you live?
    - ☐ Northern Scotland
    - ☐ Southern Scotland
    - ☐ North East
    - ☐ North West
    - ☐ Yorkshire and the Humber
    - ☐ East Midlands
    - ☐ West Midlands
    - ☐ East of England
    - ☐ South East
    - ☐ South West
    - ☐ London
    - ☐ North Wales
    - ☐ South Wales
    - ☐ Northern Ireland

30. Is there anything else you would like to tell us about yourself?

31. Would you like an Amazon voucher after participation in completing this form?
    - ☐ Yes please
    - ☐ No thank you

32. If you wish to hear about the next steps of this research, please leave your email address in the box below:

If yes, please provide the email address you'd like this to be sent to.

If you have been affected by any of the issues in this survey, please see the sources of support section below.

8. Sources of support.

We do not envisage any risks or lifestyle restrictions from taking part in this project but responding to this survey may have led to reflections about sensitive aspects of your experience of early parenthood. You can seek emotional support from the following sources.

Your GP, midwife or health visitor

Accident and Emergency at the nearest hospital or call 999

The Samaritans: 116 123 or email jo@samaritans.org. They are open 24 hours a day, 365 days a year, to listen to anything that is upsetting you for free and won't appear on your phone bill).

PANDAS: 0808 196 1776 (11am-10pm, a source of support specifically for post-natal mental health).

The charity Mind's pages on postnatal depression and perinatal mental health: https://www.mind.org.uk/information-support/types-of-mental-health-problems/postnatal-depression-and-perinatal-mental-health/

The NHS pages on post-natal depression: https://www.nhs.uk/conditions/post-natal-depression/

If you have any questions regarding this survey please contact our research team at neta.spiro@rcm.ac.uk.

To speak to someone outside the immediate study team or to raise a concern, please contact:

Prof Aaron Williamon (Head of the Centre for Performance Science) Royal College of Music, Prince Consort Road, London, SW7 2BS Email: aaron.williamon@rcm.ac.uk
