# Supplementary material for: The landscape of musical care during the beginning of life in the United Kingdom: a mixed-methods survey study
Source: BMC Complement Med Ther. 2025 Oct 16;25:380. doi: 10.1186/s12906-025-05014-6 (PMC12532952; doi:10.1186/s12906-025-05014-6)
Supplement: Supplementary file 4 — Additional File 4 [file 12906_2025_5014_MOESM4_ESM.pdf]

Musical Care Parents/Carers

1. Welcome and consent
2. Musical care activities
3. Experience of musical care activity
4. Increasing availability of musical care activities
5. Demographic questions
6. Further information
7. Sources of support

Spiro, N.,* Sanfilippo, K.R.M.,* Shaughnessy C., Rowles, M., Coombes E., Perkins, R., & Tredget, E., (2025)
The landscape of musical care during the beginning of life in the United Kingdom: A mixed-methods survey study,
BMC Complementary Medicine and Therapies. doi: 10.1186/s12906-025-05014-6

1. Welcome and consent

Thank you for considering taking part in our research. We are interested in awareness of, access to, and experience of musical care activities while families are expecting a baby and during their baby's/babies' first 2 years.

By musical care activities, we mean music listening (e.g., listening via a personal device, attending a live show) as well as music making (e.g., group music making, singing lullabies at bedtime, music classes for babies) that support people's needs and provide them with care. This could include supporting physical health or mental health, as well as relationships with other people.

In completing this survey, you will be providing valuable insight about the experience of musical care at the beginning of life and ways to increase its availability in the UK. This survey is for people who:

- currently live in the United Kingdom
- are 18 years old or over
- expectant and current parents of children born within the last 5 years.
- We are interested in hearing from mums, dads, and other caregivers.
  Please note, you may leave and return to complete the survey later on the same device.

[Participants who fully complete the survey will be entered into a prize draw for a £20 Amazon gift voucher.]

Consent
Your participation in this research is voluntary, and you may withdraw from the study at any time if you wish.

What will we do with your data?
The data you provide will be anonymous (separated from your name) and confidential (not disclosed to anyone else). The data will be stored securely at Imperial College London and the Royal College of Music according to the Colleges' Data Retention Policies. It will be used as part of the *Musical care during the beginning of life* project which is funded by the UK Research and Innovation fund to explore the development and upscaling of musical care during the beginning of life in the UK. We plan to publish portions of our data set, reports, articles, and presentations based on our findings, but you will not be identifiable from the data.

Who should I contact if I have queries about the research?
If you have any queries or concerns about the research, you can contact our research team at neta.spiro@rcm.ac.uk. The project has been reviewed and approved by the Conservatoires UK Research Ethics Committee.

We will be asking you questions about your experiences of expecting a baby and being a parent of a young baby, your mental health and wellbeing. At the end of the questionnaire, there will be links to sources of information and support on these issues.

1.1 Please confirm that you are 18 years or older and live in the UK.

- Yes, I am 18 years or older and live in the UK.

1.2 I agree to take part in the above-named project.

- Yes, I agree
- No, I do not agree

{Skip To: End of Survey If 1.2 = No, I do not agree}

2. Musical care activities

We are interested in finding out about your awareness, access to, and experience of musical care activities for people who are expecting their baby/babies and during their baby's/babies' first 2 years of life.

In this section we will ask about musical care activities that you have heard about for three groups of people:

- parent(s)/care giver(s) expecting babies (e.g., pregnant people or their partner(s), adopting parent(s), intended parent)
- parent(s)/care giver(s) with babies
- babies

2.1 (Filter question 1) Have you **heard** of any formal musical care activities for **parent(s)/care giver(s) expecting babies** (e.g., pregnant people or their partner(s), adopting parent(s), intended parent)? Here are some examples of what we mean.
- Antenatal sessions that involve some music
- Choirs/singing groups for expectant parent(s)/care giver(s)
- Dance sessions for expectant parent(s)/care giver(s)
- Live music playing in hospital for expectant parent(s)/care giver(s)
- Music classes/groups for expectant parent(s)/care giver(s)
- Music therapy groups/individual sessions for expectant parent(s)/care giver(s)
- Music therapy in hospital for expectant parent(s)/care giver(s)
- Song writing/creative sessions for expectant parent(s)/care giver(s)
- Other musical care activity like those listed here

- Yes
- No

{If 2.2 = Yes}

2.2 Please select all the formal musical care activities for **parent(s)/care giver(s) expecting babies** (e.g., pregnant people or their partner(s), adopting parent(s), intended parent) you have **heard** about.
  - Antenatal sessions that involve some music
  - Choirs/singing groups for expectant parent(s)/care giver(s)
  - Dance sessions for expectant parent(s)/care giver(s)
  - Live music playing in hospital for expectant parent(s)/care giver(s)
  - Music classes/groups for expectant parent(s)/care giver(s)
  - Music therapy groups/individual sessions for parent(s)/care giver(s)
  - Music therapy in hospital for expectant parent(s)/care giver(s)
  - Song writing/creative sessions for expectant parent(s)/care giver(s)
  - Other (please give details below) ____

2.3 Did you participate in [answer(s) selected in 2.2]?
- Yes
- No

{Skip To: 2.5 If 2.4 = Yes}

2.4 Was [answer(s) selected in 2.2] available to you?
- Yes
- No

{Skip To: 2.6 If 2.4 = No}

2.5 How did you hear about [answer(s) selected in 2.2]? (Tick all that apply)

- Class finding app/website
- Daycare
- Email mailing list
- Flyer
- GPs
- Post
- School/nursery
- Social media
- Sure start centre
- Word of mouth
- Other ____

2.6 While you or your partner were expecting a baby/babies did you do any of the follow activities *because* you were expecting? (Please tick all that apply)

- Informally sing or make music. If yes, please explain why (optional) ____
- Listen to music (on a personal device/at home). If yes, please explain why (optional) ____
- Listen to music at a formal event, e.g., a concert. If yes, please explain why (optional) ____
- None of the above

2.7 (Filter Question 2) Have you **heard** of any formal musical care activities for **parent(s)/care giver(s) with babies** up to the age of 2 years? Here are some examples of what we mean.

- Choirs/singing groups for parent(s)/care giver(s)
- Dance sessions for parent(s)/care giver(s)
- Live music playing in hospital for parent(s)/care giver(s)
- Music classes for parent(s)/care giver(s)
- Music therapy groups/individual sessions for parent(s)/care giver(s)
- Music therapy in hospital for parent(s)/care giver(s)
- Play and development groups that involve some music for parent(s)/care giver(s)
- Song writing/creative sessions for parent(s)/care giver(s)
- Other musical care activity like those listed here

- Yes
- No

{If 2.8 = Yes}

2.8 Please select all the formal musical care activities designed **for parent(s)/care giver(s) with babies** up to the age of 2 years you have **heard** about.

a) Choirs/singing groups for parent(s)/care giver(s)
b) Dance sessions for parent(s)/care giver(s)
c) Live music playing in hospital for parent(s)/care giver(s)
d) Music classes for parent(s)/care giver(s)
e) Music therapy groups/individual sessions for parent(s)/care giver(s)
f) Music therapy in hospital for parent(s)/care giver(s)
g) Play and development groups that involve some music for parent(s)/care giver(s)
h) Song writing/creative sessions for parent(s)/care giver(s)
i) Other (please give details below) ____

2.9 Did you participate in [answer(s) selected in 2.8]?

- Yes
- No

{Skip To: 2.11 If 2.9 = Yes}

2.10 Was [answer(s) selected in 2.8] available to you?

- Yes
- No

{Skip To: End of Block If 2.10 = No}

2.11 How did you hear about [answer(s) selected in 2.8]? (Tick all that apply)

- Class finding app/website
- Daycare
- Email mailing list
- Flyer
- GPs
- Post
- School/nursery
- Social media
- Sure start centre
- Word of mouth
- Other ____

2.12 While your baby/babies were up to the age of two, did you do any of the following activities <u>for yourself</u> *because* you had babies at that age? (tick all that apply)

- Informally sing or make music. If yes, please explain why (optional) ____
- Listen to music (on a personal device/at home). If yes, please explain why (optional) ____
- Listen to music at a formal event, e.g., a concert. If yes, please explain why (optional) ____
- None of the above

2.13 (Filter Question 3) Have you **heard** of any formal musical care activities designed for **<u>babies</u>** up to the age of 2 years? Here are some examples of what we mean.
- Baby massage that involves some music
- Baby yoga that involves some music
- Dance sessions for babies
- Live music playing in hospital for babies
- Music sessions for babies
- Music therapy groups/individual sessions for babies
- Music therapy in hospital for babies
- Play and development groups for babies that involve some music
- Other musical care activity like those listed here

- Yes
- No

{If 2.13 = Yes}

2.14 Please select all the formal musical care activities designed for **<u>babies</u>** up to the age of 2 years you have **heard** about.

- Baby massage that involves some music
- Baby yoga that involves some music
- Dance sessions for babies
- Live music playing in hospital for babies
- Music sessions for babies
- Music therapy groups/individual sessions for babies
- Music therapy in hospital for babies
- Play and development groups for babies that involve some music
- Other (please give details below) ____

2.15 Did you participate in [answer(s) selected in 2.14]?

- Yes
- No

{Skip To: 2.17 If 2.15 = Yes}

2.16 Was [answer(s) selected in 2.14] available to you?

- Yes
- No

{Skip To: 2.18 If 2.16 = No}

2.17 How did you hear about [answer(s) selected in 2.14]? (Tick all that apply)

- Class finding app/website
- Daycare
- Email mailing list
- Flyer
- GPs
- Post
- School/nursery
- Social media
- Sure start centre
- Word of mouth
- Other (please give details below) ____

2.18 While your baby/babies were up to the age of 2 did you do any of the following activities <u>for your baby/babies</u>? (Tick all that apply)

- Informally sing or make music. If yes, please explain why (optional)
  ____
- Listen to music (on a personal device/at home). If yes, please explain why (optional)
  ____
- Listen to music at a formal event, e.g., a concert. If yes, please explain why (optional)
  ____
- None of the above

2.19 Now that we've asked you some questions about this, do you feel that there are any musical care activities that we are missing?

____
____
____
____
____

{Display this question: If Filter Question 2.1 = No, And Filter Question 2.7 = No, And Filter Question 2.13 = No}

2.20 Would you have liked to participate in any formal musical care activities while you were expecting your baby/babies and during their baby's/babies' first 2 years of life?

(As a reminder "formal musical care activities" could include music classes/groups for expectant parents/care givers, live music playing in hospital for parents/caregivers, and baby yoga that involves some music.)

- Yes
- No

{Skip To: 2.24 If 2.8 = No}
{Display this question: If Filter Question 2.1 = Yes, And Filter Question 2.7 = Yes, And Filter Question 2.13 = Yes}

2.21 Would you have liked to participate in any **more** formal musical care activities while you were expecting your baby/babies and during their baby's/babies' first 2 years of life?

(As a reminder "formal musical care activities" could include music classes/groups for expectant parents/care givers, live music playing in hospital for parents/caregivers, and baby yoga that involves some music.)

- Yes
- No

{Skip To: 2.24 If 2.8 = No}

{Display this question: If 2.20 = Yes, Or 2.21 = Yes}

2.22 What do you think stood in your way of participating in more musical care activities? (Tick all that apply)

- I didn't feel they were open to me
- I didn't have a way of getting to activities
- I didn't have access to musical equipment
- I didn't have confidence in my musical abilities
- I didn't have confidence using digital equipment (e.g., laptop for joining activities online)
- I didn't have the money
- I didn't have time
- I had issues around childcare (e.g., an older child to care for)
- I later found out about activities that I could have gone to
- There was a language barrier
- There weren't any activities available
- Other (please give details below) ____

{Display this question: If 2.20 = Yes, Or 2.21 = Yes)

2.23 What would support you to participate in such activities?

____
____
____
____
____

{Display this question: If 2.20 = No, Or 2.21 = No}

2.24 Please describe why not.

____
____
____
____
____

2.25 If you were to participate in a musical care activity, how important would the following elements be?

	very important	important	neither important nor unimportant	not very important	not important
Delivery method (e.g., virtual, in person)					
Group make-up (father's only group, mum's other group, etc.)					
Group size					
Inclusivity and feeling welcome					
Location (e.g., distance from home)					
Own confidence in musical abilities					
Pram access					
Recommendation from others					
Training/credentials of group leader					
Value for money					

3. Experience of musical care activity

3.1 Please think about a specific musical care activity that you've done that was the most memorable to you (whether for good or bad reasons).

Focusing on that experience

...could you describe the activity?

____
____
____
____
____

3.2 ...could you tell us about what it was like for you?

____
____
____
____
____

3.3 When I took part in the activity...

- My partner or I were pregnant/expecting a baby
- I participated with my child/children
- My partner or I were pregnant/expecting a baby and I participated with my child/children

3.4 How old was/were your baby/babies when you started the activity?

- 0 - 6 months
- 6 - 12 months
- 12 months - 18 months
- 18 months - 24 months

3.5 For how long did you take part in this activity?

- 1 day
- 1 week - 3 weeks
- 1 month - 2 months
- 3 months - 6 months
- 6 months - 1 year
- More than 1 year

3.6 How frequently do/did you take part in this activity?

- Daily
- Weekly
- Every other week
- Monthly
- Every other month
- Once

3.7 Why did you want to take part in this particular activity?

____
____
____
____
____

3.8 How did you hear about it? (Tick all that apply)

- ○ Class finding App/website
- ○ Daycare
- ○ Email mailing list
- ○ Flyer
- ○ GPs
- ○ Post
- ○ School/nursery
- ○ Social media
- ○ Sure start centre
- ○ Word of mouth
- ○ Other (please give details below) ____

3.9 How was it described to you when you heard about it? (e.g., as a music class for mums and babies or as a singing group for pregnant women, or a father-and-baby group)

____
____
____
____
____

3.10 Please indicate below whether or not participation in the musical care activity affected you in these ways:

	I felt much more...	I felt a little more...	I felt just as...	I felt a little less...	I felt much less...	Not applicable
Anxious						
Close to my baby						
Close to my partner (if applicable)						
Confident						
Connected to other people						
Depressed						
Happy						
Lonely						
Relaxed						
Worried						
Other (please describe below)						

3.11 Please indicate below whether or not you agree/disagree with the following statements about your participation in the musical care activity:

	strongly agree	agree	neither agree nor disagree	disagree	strongly disagree
I did something for my baby/child					
I did something for myself					
I did something new					
I didn't get anything out of it					
I didn't like it					
I got to know and understand my baby better					
I learnt new music					
I met new friends					
I saw a different side to my baby					
Other (please describe below)					

3.12 Would you recommend this activity to others?

	strongly recommend	recommend	neither recommend nor not recommend	do not recommend	strongly not recommend
Would you recommend this activity to others?					

3.13 What was the cost of this activity per session?

- Free
- Less than £5
- £5 - £10
- £10 - £15
- More than £15

3.14 Are/is there any skills/knowledge that you have gained through taking part in the musical care activity that you continue to use?

____
____
____
____
____

3.15 If you know it, please share the website or online information about this musical care activity? (e.g., a Facebook page or a website describing the activity).

____
____
____
____
____

3.16 If you take part in musical care activity groups, do you find that these groups are diverse in terms of who is there?

	very diverse	diverse	neither diverse nor not diverse	not very diverse	not diverse at all
How diverse are these groups?					

3.17 What do you think we can do to improve the diversity of musical care activity groups, if anything at all?

____
____
____
____
____

4. Increasing availability of musical care activities

4.1 Do you feel that we should increase the availability of musical care activities for **parent(s)/care giver(s) expecting babies**?

- Yes
- No

{Display this question: If 4.1 = Yes}

4.2 Please choose the 3 activities for **parent(s)/care giver(s) expecting babies** that you would like to see more of. Please rank these from most important to see more of to least (1 = most, 3 = least).
_______ Antenatal sessions that involve some music
_______ Choirs/singing groups for expectant parents/care givers
_______ Dance sessions for expectant parents/care givers
_______ Live music playing in hospital for expectant parents/care givers
_______ Music classes/groups for expectant parents/care givers
_______ Music therapy groups/individual sessions for expectant parents/care givers
_______ Music therapy in hospital for expectant parents/care givers
_______ Song writing/creative sessions for expectant parents/care givers

4.3 Do you feel that we should increase the availability of musical care activities for **parents/care givers with babies** up to the age of 2 years?

- Yes
- No

{Display this question: If 4.3 = Yes}

4.4 Please choose the 3 activities for **parents/care givers with babies** up to the age of 2 years that you would like to see more of. Please rank these from most important to see more of to least (1 = most, 3 = least).
_______ Choirs/singing groups for parents/caregivers
_______ Dance sessions for parents/caregivers
_______ Live music playing in hospital for parents/caregivers
_______ Music classes for parents/caregivers
_______ Music therapy groups/individual sessions for parents/caregivers
_______ Music therapy in hospital for parents/caregivers
_______ Play and development groups that involve some music for parents/caregivers
_______ Song writing/creative sessions for parents/caregivers

4.5 Do you feel that we should increase the availability of musical care activities for **babies** up to the age of 2 years?

- Yes
- No

{Skip To: 4.8 If 4.5 = No}
{If 4.5 = Yes)

4.6 Please choose the 3 activities for **babies** up to the age of 2 years that you would like to see more of. Please rank these from most important to see more of to least (1 = most, 3 = least).
_______ Baby massage that involves some music
_______ Baby yoga that involves some music
_______ Dance sessions for babies

_______ Live music playing in hospital for babies
_______ Music sessions for babies
_______ Music therapy groups/individual sessions for babies
_______ Music therapy in hospital for babies
_______ Play and development groups for babies that involve some music

4.7 Are there any other activities that you would like to see more of that you would rank in the top 3?

- Yes (if yes, which activities) ____
- No

4.8 Do you feel that there are barriers to accessing musical care activities?

- Yes (If yes, what barriers are there?) ____
- No

4.9 Is there anything else you would like to tell us about musical care during the beginning of life?

____
____
____
____
____

5. Demographic questions

5.1 In which region(s) do you live?

- Highlands and Islands
- Northern Scotland
- Southern Scotland
- North East England
- North West England
- Yorkshire and the Humber
- East Midlands
- West Midlands
- East of England
- South East England
- South West England
- London
- North Wales
- Mid Wales
- West Wales
- South Wales
- Northern Ireland
- Would rather not say

5.2 How old are you?
*Please write in numbers, e.g., 42*____

5.3 I identify myself as

- Female
- Male
- Non-binary
- Would rather not say
- Other (please define) ____

5.4 Relationship status:

- Single
- Married or domestic partnership
- Widowed
- Divorced
- Separated
- Other
- Would rather not say

5.5 Do you consider yourself to have a disability as defined by the Equality Act 2010. (The Equality Act 2010 defines a disabled person as someone who has a physical or mental impairment which has a substantial and adverse long-term effect on his or her ability to carry out normal day-to-day activities)

- Yes
- No
- Would rather not say

5.6 I classify myself as (please tick all that apply):
This list is adapted from the Office for National Statistics. Different countries and groups use different labels, so please add your own if you wish.

- White - English / Welsh / Scottish / Northern Irish / British
- White - Irish
- White - Gypsy or Irish Traveller
- Any other White Background
- Mixed / Multiple ethnic groups - White and Black Caribbean
- Mixed / Multiple ethnic groups - White and Black African
- Mixed / Multiple ethnic groups - White and Asian
- Any other Mixed / Multiple ethnic background
- Asian / Asian British - Indian
- Asian / Asian British - Pakistani
- Asian / Asian British - Bangladeshi
- Asian / Asian British - Chinese
- Any other Asian background
- Black / African / Caribbean / Black British - African
- Black / African / Caribbean / Black British - Caribbean
- Any other Black / African / Caribbean background
- Arab
- Any other ethnic group ____
- Would rather not say

5.7 Do you work in music professionally?
*By **professional** we mean that some part, or all, of your livelihood comes from work in one or more arts and cultural areas.*

- Yes
- No

5.8 Approximately, what is your yearly household income (excluding parental leave periods)?

- Unemployed/Full-time student
- Retired
- Less than £10,000
- £10,000-£19,000
- £20,000-£29,000
- £30,000-£39,000
- £40,000-£49,000
- £50,000-£59,000
- £60,000-£70,000
- More than £70,000
- Would rather not say

5.9 What is the highest educational and/or vocational qualification you have already attained?

- Did not complete any school qualification
- Completed first school qualification at about 16 years (e.g., GCSE)
- Completed second qualification (e.g,. A levels/BTEC/High School)
- Completed first school qualification at about 16 years (e.g., GCSE)
- Undergraduate degree or professional qualification (e.g., bachelors degree/NVQ 6)
- Postgraduate degree (e.g., masters, PHD, DMA, DMus degree, NVQ7)
- I am still in education

{If 5.9 = I am still in education}

5.10 If you are still in education, what is the highest qualification you expect to obtain?

- First School qualification (e.g., GCSE)
- Post-16 vocational course (e.g., Apprenticeship)
- Second school qualification (e.g., A levels/High School)
- Undergraduate degree or professional qualification (e.g., bachelors degree/NVQ 6)
- Postgraduate degree (e.g., masters, PHD, DMA, DMus degree, NVQ7)
- Not applicable

5.11 Are you currently on parental leave?

- Yes
- No

5.12 How many children do you have?
(Please write in numbers, e.g., 3) ____

5.13 Are you or your partner currently pregnant/expecting a baby?

- Yes
- No

5.14 With whom do you live? (Please tick all that apply)

- On my own
- My spouse or partner
- Extended family (e.g., parents, siblings)
- Friends or house share
- Other (please describe below) ____
- Would rather not say

5.15 We'd like to learn about your experience with music. Please select the most appropriate category:

	completely agree	strongly agree	agree	neither agree nor disagree	disagree	strongly disagree	completely disagree
I spend a lot of my free time doing music-related activities.							
I enjoy writing about music, for example on blogs and forums.							
If somebody starts singing a song I don't know, I can usually join in.							
I can sing or play music from memory.							
I am able to hit the right notes when I							

sing along with a recording.
I can compare and discuss differences between two performances or versions of the same piece of music.
I have never been complimented for my talents as a musical performer.
I often read or search the internet for things related to music.
I am not able to sing in harmony when somebody is singing a familiar tune.
I am able to identify what is special about a given musical piece.
When I sing, I have no idea whether I'm in tune or not.
Music is kind of an addiction for me - I couldn't live without it.
I don’t like singing in public because I’m afraid that I would sing wrong notes.
I would not consider myself a musician.
After hearing a new song two or three times, I can usually sing it by myself.

5.16 Please select the most appropriate category:

	0	1	2	3	4-5	6-9	10 or more
I engaged in regular, daily practice of a musical instrument (including voice) for ___ years.							

5.17 Please select the most appropriate category:

	0	0.5	1	1.5	2	3-4	5 or more
At the peak of my interest, I practiced ___ hours per day on my primary instrument.							

5.18 Please select the most appropriate category:

	0	1	2	3	4	5	6 or more
I can play ___ musical instruments							

6. Further information

Thank you for your help!

If you have been affected by any of the issues in this survey, please see the sources of support section below.

If you wish to participate in the next parts of this research, please leave your email address here. (Your email address will not be linked to your data):

____

If you would like to be entered into a prize draw for a £20 Amazon gift voucher, please leave your email address here. (Your email address will not be linked to your data).

____

7. Sources of support

We do not envisage any risks or lifestyle restrictions from taking part in this project but responding to this survey may have led to reflections about sensitive aspects of your experience of early parenthood. You can seek emotional support from the following sources.

- Your GP, midwife or health visitor
- Accident and Emergency at the nearest hospital or call 999
- The Samaritans: 116 123 or email jo@samaritans.org. They are open 24 hours a day, 365 days a year, to listen to anything that is upsetting you for free and won't appear on your phone bill).
- PANDAS: 0808 196 1776 (11am-10pm, a source of support specifically for post-natal mental health).
- The charity Mind's pages on postnatal depression and perinatal mental health: https://www.mind.org.uk/information-support/types-of-mental-health-problems/postnatal-depression-and-perinatal-mental-health/
- The NHS pages on post-natal depression: https://www.nhs.uk/conditions/post-natal-depression/
- This NHS link provides information and processes for seeking support related to Coronavirus (COVID-19).

If you have any questions regarding this survey, please contact our research team at neta.spiro@rcm.ac.uk.
Please click the FORWARD arrow button to submit your response.
