# Supplementary material for: The landscape of musical care during the beginning of life in the United Kingdom: a mixed-methods survey study
Source: BMC Complement Med Ther. 2025 Oct 16;25:380. doi: 10.1186/s12906-025-05014-6 (PMC12532952; doi:10.1186/s12906-025-05014-6)
Supplement: Supplementary file 5 — Additional File 5 [file 12906_2025_5014_MOESM5_ESM.pdf]

Musical care activity providers

1. Welcome and consent
2. Musical care activities
3. Increasing availability of musical care activities
4. Demographic questions
5. Further information
6. Sources of support

Spiro, N.,* Sanfilippo, K.R.M.,* Shaughnessy C., Rowles, M., Coombes E., Perkins, R., & Tredget, E., (2025) The landscape of musical care during the beginning of life in the United Kingdom: A mixed-methods survey study, *BMC Complementary Medicine and Therapies*. doi: 10.1186/s12906-025-05014-6

1. Welcome and consent

Thank you for considering taking part in our research. We are interested in your work in musical care activities with people expecting a baby and/or during their baby's/babies' first 2 years. By completing this survey you will be providing valuable insight about the experience of musical care at the beginning of life and ways to increase its availability in the UK.

This survey is for people who:

- Work in musical care in the UK
- Currently live in the United Kingdom
- Are 18 years old or over

Please note, you may leave and return to complete the survey later on the same device.

Participants who fully complete this survey will be entered into a draw for a 20 pounds Amazon gift voucher.

Consent
Your participation in this research is voluntary, and you may withdraw from the study at any time if you wish.

What will we do with your data?
The data you provide will be anonymous (separated from your name) and confidential (not disclosed to anyone else). The data will be stored securely at Imperial College London and the Royal College of Music according to the Colleges' Data Retention Policies. It will be used as part of the *Musical care during the beginning of life* project which is funded by the UK Research and Innovation fund to explore the development and upscaling of musical care during the beginning of life in the UK. We plan to publish portions of our data set, reports, articles, and presentations based on our findings, but you will not be identifiable from the data.

Who should I contact if I have queries about the research? If you have any queries or concerns about the research, you can contact our research team at neta.spiro@rcm.ac.uk. The project has been reviewed and approved by the Conservatoires UK Research Ethics Committee.

1.1 Please confirm that you are 18 years or older and live in the UK.

- Yes, I am 18 years or older and live in the UK

1.2 Please confirm that you give your informed consent to participate in the study

- Yes, I agree
- No, I do not agree

{Skip To: End of Survey If 1.2 = No, I do not agree}

2. Musical care activities

We are interested in finding out about awareness of, access to, and experience of musical care activities in which people participate while they are expecting their baby/babies and during their baby's/babies' first 2 years of life.

By musical care activities, we mean music listening (e.g., listening via a personal device, attending a live show) as well as music making (e.g., group music making, singing lullabies at bedtime, music classes for babies) that support people's needs and provide them with care. This could include supporting physical health or mental health, as well as relationships with other people.

2.1 What musical care activities for parent(s)/care giver(s) expecting babies (e.g., pregnant people or their partner(s), adopting parent(s), intended parent) do you currently deliver / have you delivered in the last 5 years? (Tick all that apply)

- None
- Antenatal sessions that involve some music
- Choirs/singing groups for expectant parent(s)/care giver(s)
- Dance sessions for expectant parent(s)/care giver(s)
- Live music playing in hospital for expectant parent(s)/care giver(s)
- Music classes/groups for expectant parent(s)/care giver(s)
- Music therapy groups/individual sessions for expectant parent(s)/care giver(s)
- Music therapy in hospital for expectant parent(s)/care giver(s)
- Song writing/creative sessions for expectant parent(s)/care giver(s)
- Other (please give details below)

  ____

2.2 What musical care activities designed for parent(s)/care giver(s) with babies up to the age of 2 years do you currently deliver / have you delivered in the last 5 years? (Tick all that apply)

- None
- Choirs/singing groups for parent(s)/caregiver(s)
- Dance sessions for parent(s)/caregiver(s)
- Live music playing in hospital for parent(s)/caregiver(s)
- Music classes for parent(s)/caregiver(s)
- Music therapy groups/individual sessions for parent(s)/caregiver(s)
- Music therapy in hospital for parent(s)/caregiver(s)
- Play and development groups that involve some music for parent(s)/caregiver(s)
- Song writing/creative sessions for parent(s)/caregiver(s)
- Other (please give details below)

  ____

2.3 What musical care activities designed for babies up to the age of 2 years do you currently deliver / have you delivered in the last 5 years? (Tick all that apply)

- None
- Baby massage that involves some music
- Baby yoga that involves some music
- Dance sessions for babies
- Live music playing in hospital for babies
- Music sessions for babies
- Music therapy groups/individual sessions for babies
- Music therapy in hospital for babies
- Play and development groups for babies that involve some music
- Other (please give details below)

  ____

2.4 Please think about a specific musical care activity that you deliver or have delivered in the last 5 years.

Focusing on that activity, could you

...describe that activity?

____
____
____
____
____

2.5 ...tell us about the aim of the activity?

____
____
____
____
____

2.6 ...tell us who is / was invited to take part? (e.g., mums, dads, babies with special needs, mums with post-natal depression)

____
____
____
____
____

2.7 ...tell us the demographic characteristics of the people who usually participate / participated? (e.g., ages, cultural background, languages spoken at home, socio-economic background, physical and mental capacities, gender, etc.)

____
____
____
____
____

2.8 How is / was this activity advertised? (Tick all that apply)

- ○ Class finding app/website
- ○ Daycare
- ○ Email mailing list
- ○ Flyer
- ○ GPs
- ○ Post
- ○ School/nursery
- ○ Social media
- ○ Sure start centre
- ○ Word of mouth
- ○ Other (please describe below)

  ____

2.9 Do / did you evaluate the success of this activity?

- Yes (If yes, how?) (e.g., Personal accounts, Internal evaluation, External formal evaluation)
  ____
- No

{If 2.9 = Yes}

2.10 Can you share any of these evaluations/research? (By sharing a link here, a short summary, or, if you prefer, emailed to the research team at neta.spiro@rcm.ac.uk)

____
____
____
____
____

{Display this question: If 2.9 = Yes}

2.11 Please describe how you fund / funded this activity.

____
____
____
____
____

{Display this question: If 2.9 = Yes}

2.12 Have you had any education/training or other experience(s) in preparation for delivering this activity?

- Yes (If yes, please describe below) ____
- No

{Display this question: If 2.9 = No}

2.13 Can you share any online materials about the activity (e.g., a website, facebook page).

____
____
____
____
____

3. Increasing availability of musical care activities

3.1 What are the barriers, if any, that prevent you from delivering musical care activities in the ways you would like? (e.g., training, access, resources, cultural barriers, time). If there are no barriers please write "no barriers".

____
____
____
____
____

3.2 What enables you, if anything, to deliver musical care activities in the ways you would like? (e.g., training, access, resources, cultural awareness, time)

____
____
____
____
____

3.3 Are any grants or sources funding available to you?

- Yes (If yes, please describe) ____
- No

3.4 Do you take part in any continuing professional development / training?

- Yes (If yes, please describe) ____
- No

3.5 Do you feel there are barriers to accessing training opportunities? (e.g., cost/location/relevance/quality)

- Yes (If yes, please describe) ____
- No

3.6 Do you see a need to diversify the range of people who attend your musical care activities?

- Yes
- No

{Display this question: If 3.6 = Yes}

3.7 Have you tried to diversify who attends?

- Yes (If yes, what did you do?) ____
- No

{Display this question: If 3.7 = Yes}

3.8 What worked and what didn't work to diversify who attends?

____
____
____
____
____

{Display this question: If 3.7 = Yes}

3.9 Is there anything you would have liked/or would like to do to diversify who attends?

- Yes (If yes, what would you do and what would you need to be able to do it?)
  ____
- No

3.10 How can the musical workforce build capacity in this area of work? (e.g., Upskilling/training providers, allocation of resources)

____
____
____
____
____

3.11 Who are the stakeholders we need to gain support from in order to scale up musical care activities during the beginning of life?

____
____
____
____
____

3.12 Can you tell us a little about what drew you to work in the area of musical care during the beginning of life?

____
____
____
____
____

3.13 Is there anything else you would like to tell us about musical care during the beginning of life?

____
____
____
____
____

4. Demographic questions

4.1 In which region(s) do you live?

- o Highlands and Islands
- o Northern Scotland
- o Southern Scotland
- o North East England
- o North West England
- o Yorkshire and the Humber
- o East Midlands
- o West Midlands
- o East of England
- o South East England
- o South West England
- o London
- o North Wales
- o Mid Wales
- o West Wales
- o South Wales
- o Northern Ireland
- o Would rather not say

4.2 How old are you?
Please write in numbers, e.g., 42 ____

4.3 I identify myself as

- Female
- Male
- Non-binary
- Would rather not say
- Other (please define) ____

4.4 I classify myself as (please tick all that apply):
This list is adapted from the Office for National Statistics. Different countries and groups use different labels, so please add your own if you wish.

- White - English / Welsh / Scottish / Northern Irish / British
- White - Irish
- White - Gypsy or Irish Traveller
- Any other White Background
- Mixed / Multiple ethnic groups - White and Black Caribbean
- Mixed / Multiple ethnic groups - White and Black African
- Mixed / Multiple ethnic groups - White and Asian
- Any other Mixed / Multiple ethnic background
- Asian / Asian British - Indian
- Asian / Asian British - Pakistani
- Asian / Asian British - Bangladeshi
- Asian / Asian British - Chinese
- Any other Asian background
- Black / African / Caribbean / Black British - African
- Black / African / Caribbean / Black British - Caribbean
- Any other Black / African / Caribbean background
- Arab
- Any other ethnic group
- Would rather not say

4.5 What is the highest educational and/or vocational qualification you have attained?

- Did not complete any school qualification
- Completed first school qualification at about 16 years (e.g., GCSE)
- Completed second qualification (e.g., A levels/BTEC/High School)
- Completed first school qualification at about 16 years (e.g., GCSE)
- Undergraduate degree or professional qualification (e.g., bachelors degree/NVQ 6)
- Postgraduate degree (e.g., masters, PHD, DMA, DMus degree, NVQ7)
- I am still in education

4.6 If you are still in education, what is the highest qualification you expect to obtain?

- First School qualification (e.g., GCSE)
- Post-16 vocational course (e.g., Apprenticeship)
- Second school qualification (e.g., A levels/BTEC/High School)
- Undergraduate degree or professional qualification (e.g., bachelors degree/NVQ 6)
- Postgraduate degree (e.g., masters, PHD, DMA, DMus degree, NVQ7)
- Not applicable

4.7 Do you have training in delivering musical care activities, in the perinatal period, or in early years work? (e.g., a masters in music therapy, short courses in community music making, undergraduate degree in child development)?

- Yes (If so, what was it?)____
- No

4.8 Do you consider yourself to have a disability as defined by the Equality Act 2010? (The Equality Act 2010 defines a disabled person as someone who has a physical or mental impairment which has a substantial and adverse long-term effect on his or her ability to carry out normal day-to-day activities)

- Yes
- No
- Would rather not say

4.9 How long have you been a musical care practitioner?

- less than 6 months
- 6 – 12 months
- 1 – 5 years
- 5 – 10 years
- 10+ years

4.10 How do you describe your musical care work? (e.g., Music therapy, Music education, Music entertainment, Community music)

____
____
____
____
____

4.11 Is delivering musical care activities your primary source of income?

- Yes
- No (If no, what is your primary source of income?)
  ____

4.12 Approximately, what is your yearly household income?

- Unemployed/Full-time student
- Retired
- Less than £10,000
- £10,000-£19,000
- £20,000-£29,000
- £30,000-£39,000
- £40,000-£49,000
- £50,000-£59,000
- £60,000-£70,000
- More than £70,000
- Would rather not say

5. Further information

Thank you for your help!

If you have been affected by any of the issues in this survey, please see the sources of support section below.

If you wish to participate in the next parts of this research, please leave your email address here. (Your email address will not be linked to your data).
____

If you would like to be entered into a draw for a 20 pounds Amazon voucher, please leave your email address here. (Your email address will not be linked to your data).
____

6. Sources of support

We do not envisage any risks or lifestyle restrictions from taking part in this project but responding to this survey may have led to reflections about sensitive aspects of your experience of early parenthood. You can seek emotional support from the following sources.

- Your GP, midwife or health visitor
- Accident and Emergency at the nearest hospital or call 999
- The Samaritans: 116 123 or email jo@samaritans.org. They are open 24 hours a day, 365 days a year, to listen to anything that is upsetting you for free and won't appear on your phone bill).
- PANDAS: 0808 196 1776 (11am-10pm, a source of support specifically for post-natal mental health).
- The charity Mind's pages on postnatal depression and perinatal mental health: https://www.mind.org.uk/information-support/types-of-mental-health-problems/postnatal-depression-and-perinatal-mental-health/
- The NHS pages on post-natal depression: https://www.nhs.uk/conditions/post-natal-depression/
- This NHS link provides information and processes for seeking support related to Coronavirus (COVID-19).

If you have any questions regarding this survey please contact our research team at neta.spiro@rcm.ac.uk.
Please click the FORWARD arrow button to submit your responses.
