# Supplementary material for: The landscape of musical care during the beginning of life in the United Kingdom: a mixed-methods survey study
Source: BMC Complement Med Ther. 2025 Oct 16;25:380. doi: 10.1186/s12906-025-05014-6 (PMC12532952; doi:10.1186/s12906-025-05014-6)
Supplement: Supplementary file 6 — Additional File 6 [file 12906_2025_5014_MOESM6_ESM.pdf]

Additional File 6. Musical care work

How do you describe your musical care work (e.g., Music therapy, Music education, Music entertainment, Community music)?*	*n*(%)
Baby and parent/carer classes.	*1*(2)
Beautiful Experience	*1*(2)
BSL tutor teach the nursery rhymes with the music	*1*(2)
Child development using music. Preschool music classes.	*1*(2)
Community group	*1*(2)
Community music	*4*(8)
Community music, arts for personal and social benefit/change.	*1*(2)
Community musician/early years music practitioner.	*1*(2)
Community-based music	*1*(2)
Concerts for children and families - music entertainment	*1*(2)
Early years developmental sessions - community classes	*1*(2)
Entertainment and community	*1*(2)
I'm a project director/producer - not practitioner	*1*(2)
I'm an organiser / manager for programmes in health and education settings.	*1*(2)
Informal music & movement classes	*1*(2)
Learning through play	*1*(2)
Little Notes is community music - but I find it harder & harder not to infuse my musical interactions with a Therapeutic bent, so my classes are gentle & inclusive & nurturing.	*1*(2)
Music Activity	*1*(2)
Music and movement	*1*(2)
Music and movement education	*2*(4)
Music and sensory experience	*1*(2)
Music as part of play and development groups	*1*(2)
Music education	*2*(4)
Music education & community music	*1*(2)
Music Education/Community Music	*1*(2)
Music educational class	*1*(2)
Music Entertainment	*2*(4)
Music entertainment, but also education for the community :)	*1*(2)
Music facilitator	*1*(2)
Music therapy	*4*(8)
Music Therapy - community based	*1*(2)
Music therapy - community music therapy	*1*(2)
Music therapy and community music	*1*(2)
music-arts practice	*1*(2)
musical education and community music.	*1*(2)
musical treatment	*1*(2)
Non-existent at present	*1*(2)
Parent and baby classes	*1*(2)
Singing for health and wellbeing, community music leader, choir leader/facilitator	*1*(2)
Singing for well-being	*1*(2)
Singing to make you smile	*1*(2)

Spiro, N.,* Sanfilippo, K.R.M.,* Shaughnessy C., Rowles, M., Coombes E., Perkins, R., & Tredget, E., (2025) The landscape of musical care during the beginning of life in the United Kingdom: A mixed-methods survey study, *BMC Complementary Medicine and Therapies.* doi: 10.1186/s12906-025-05014-6

*As we have seen in other work in this area, when asked to describe their musical care work, people often pick more than one description and a number of closely related terms.
