# Supplementary material for: The landscape of musical care during the beginning of life in the United Kingdom: a mixed-methods survey study
Source: BMC Complement Med Ther. 2025 Oct 16;25:380. doi: 10.1186/s12906-025-05014-6 (PMC12532952; doi:10.1186/s12906-025-05014-6)
Supplement: Supplementary file 7 — Additional File 7 [file 12906_2025_5014_MOESM7_ESM.pdf]

Spiro, N.,* Sanfilippo, K.R.M.,* Shaughnessy C., Rowles, M., Coombes E., Perkins, R., & Tredget, E., (2025) The landscape of musical care during the beginning of life in the United Kingdom: A mixed-methods survey study, *BMC Complementary Medicine and Therapies*. doi: 10.1186/s12906-025-05014-6

Additional File 7. Codebook

MUSICAL CARE ACTIVITIES: Music dominant and multi-practice activities

Multi-practice activities that involves music

“... baby development group which used songs and rhymes to play games/actions etc with babies including sensory stimulation/massage/yoga etc.” Parent/Caregiver (S Wales, 30, F, White).	"Use song and rhymes to interact and develop the participation in the class. In Baby group use the same rhyme before starting so the babies become familiar with the activity such as baby massage, mother and baby yoga and Bath Babies. Supports their body awareness when doing movement or massage” Provider (Parent and baby classes, W Midlands, 39, F, White)	"I run a stay and play and for 20 minutes at the end we do themed songs depending what the theme is. We usually use different props or actions” Provider (Entertainment and community, NW England, 32, F, White)

Music groups

"It is a nanny music class which uses a mix of traditional and new songs, movement and instruments" Parent/Caregiver (SE England, 38, F, White)	“... a weekly class involving singing, exploring, finding rhythm, and making new friends” Parent/Caregiver (SW England, 37, F, White)	“Music & movement sessions which try & involve the parent/caregiver too. A mix of new & traditional songs & rhymes that encourage participants to dance/ follow actions/ play along with simple percussion instruments. We also include Makaton” Provider (Informal music and movement classes, SE England, 57, F, White)

Live concerts primarily for babies

“...concert at church - baby was very attentive to the music and the sounds" Parent/Caregiver (SE England, 50, M, White)	"Classical music in a relaxed atmosphere for babies, toddlers and young children. Concerts last 40 minutes and babies can crawl and explore whilst listening to the music" Provider (Concerts for children and families, and music entertainment, SW England, 44, F, White)

Music therapy

“Individual music therapy session with a mum and her newborn - mum suffering from severe PPD and psychosis and in a catatonic state. Helped mum to acknowledge baby by singing to him - Mum shared she used to go to church so I sang a hymn to which she spontaneously joined in.” Provider (Music Therapy, London, 38, F, White)	“Music therapy group for young homeless mothers and their babies…I have also run music therapy groups for mothers with postnatal depression and their babies, and community groups for mothers and babies” Provider (Music Therapy, funded by local charity, London, 47, F, Any other White)

PARENTS' PERSONAL AND LOGISTICAL FACTORS OF MOTIVATION AND DETERENCE

**PARENTS' MOTIVATORS**		
**Personal preference**		
"Because it represents us as a family that we not only love to spend time together, but we have a passion for music and dancing together" Parent/Caregiver (W Midlands, 36, F, White)	"My son started to dance when he heard music on the radio/tv etc. so thought I'd try a class to see if he enjoyed it" Parent/Caregiver (SE England, 30, F, White)	
**Experiential and practical factors**		
Experience something new		
"To meet other mums and let my child explore something new" Parent/Caregiver (SW England, 34, F, White)		
Get out of the house		
"To get my baby out of the house and mixing with other children" Parent/Caregiver (S Scotland, 37, F, White)		
Convenient		
"It was only £2 a session and close to my home" Parent/Caregiver (W Midlands, 28, F, White)	"It was accessible and affordable. It was located at my elder child's school so it was in a good location" Parent/Caregiver (E of England, 32, F, White)	"Because I could take both of my children" Parent/Caregiver (NE England, 33, F, White)
**Recommendation by healthcare provider**		
"My child had a speech delay so I was trying to do more activities which might help with language development and [named provider] was recommended to me by our health visitor at the time" Parent/Caregiver (SW England, 37, F, White)		
**Expectation of benefit**		
Perception that activity would be helpful		
"My partner is very musical and so wanted our son to experience it. Knows the positive effect of music" Parent/Caregiver (London, 35, M, Black/African/Caribbean/Black British)		
Be part of a community		
"I felt like it would mix things up and I would meet people like myself and possibly make friends" Parent/Caregiver (East of England, 24, M, White)	"To … be part of a group of mums for support" Parent/Caregiver (N Ireland, 36, F, White)	
Bonding		
"To bond with my baby and enjoy the experience with my wife" Parent/Caregiver (SE England, 44, M, White)		

**DETERENCE**
Resources and logistics
“…it’s very hard to get into play groups where I live, she’s 8 months old and still haven’t been accepted into one”<br>Parent/Caregiver (SE England, 22, F, White)
Inclusivity and diversity
“Make it more inclusive such as more diversity and ask if it could include songs from different cultures too”<br>Parent/Caregiver (London, 25, F, Asian)
“I suffer with anxiety so it was a struggle going new places with my baby”<br>Parent/Caregiver (NE England, 25, F, White)
“Some friendly. Others I have tried are very 'clique' and not tolerant of fathers” Parent/Caregiver (NE England, 32, F, White)

PROVIDERS’ MOTIVATIONS AND CHALLENGES

Personal experience
"My daughter is learning disabled and we were able to access music therapy when she was little. When I attended classes with my son, I thought it was a wasted opportunity to help children develop good listening skills and support parents with positive behaviour strategies."<br>Provider (Baby and parent/carer classes, SE of England, 56, F, White)
Professional experience
“I have taught music in my Primary school setting and wanted to work with babies and younger children and their parents. I firmly believe in music to support children in all areas of learning”<br>Provider (Music educational classes, NE England, 44, F, White)
Gap in the market
"Saw an opportunity to not only support new mums (having recently become a mum) but also to use LIVE music (as most toddler groups use pre-recorded music"<br>Provider (Music Therapist, E of England, 32, F, White)
Coordination and collaboration
“Training and knowledge exchange are needed for music educators and musicians to understand the best ways to work in early childhood.<br>Provider (Music-arts practice, E of England, 63, F, White)

SEEING INDIVIDUAL AND SOCIAL EXPERIENCES AND OUTCOMES

**Parent/caregiver Outcomes**	
Learning and gaining confidence	
“It was interactive, taught me a lot of how to play with baby in the early days and I got to know lots of the other parents” Parent/Caregiver (S Scotland, 35, F, White)	"To encourage interaction and confidence in communication with mother and baby" Provider (Music Therapist, London, 47, F, Any other white background)
Building a community	
“It was a lovely experience and the same parents would be there each week so we managed to build little friendships” Parent/Caregiver (NE England, 37, F, White)	"To offer support and develop peer relationships." Provider (Music Therapist, London, 47, F, Any other white background)
Enjoyment	
"The session was organised perfectly which made the experience full of joy” Parent/Caregiver (W Midlands, 37, M, Asian – Asian British /Pakistani)	"To have fun with music" Provider (Informal music and movement classes, SE England, 57, F, White)
Relaxation	
"I enjoyed the experience a lot, I felt at ease and comforted" Parent/Caregiver (W Midlands, 37, M, Asian – Asian British /Pakistani)	"To ... relax both parents and baby" Provider (Parent and baby classes, W Midlands, 39, F, White)
Support mental health	
“Individual music therapy session with a mum and her newborn - mum suffering from severe PPD [post-partum depression] and psychosis and in a catatonic state. Helped mum to acknowledge baby by singing to him - Mum shared she used to go church so I sang a hymn to which she spontaneously joined in” Provider (Music Therapy, London, 38, F, White,)	"When my wife was pregnant we went to a music therapy type group for expectant mums, purely as my wife was stressed a lot, the music was the reason that all mums went" Parent/caregiver (East of England, 36, M, White)
**Infant Outcomes**	
Enjoyment and play	
"I remember feeling a bit embarrassed but my baby loved it which is the main thing” Parent/Caregiver (SW England, 28,F, White)	"Fun and learning through play and music" Provider (Music and sensory experience, F, 43, Any Other White)
Engagement and socialising	
"Sharing natural voice songs, nursery rhymes, using music to move and reenact a story...to engage the babies, children and parents/care-givers in music, storytelling and singing" Provider (Community music, Arts for personal and social benefit/change, 40, F, White)	“It was lovely to be out with the baby and being able to socialise with other parents plus I felt it was really stimulating him” Parent/Caregiver (NW England, 44, F, White,)

Relaxation		
“It was enjoyable to see how my baby reacted to the singing and to help them relax”<br>- Parent/Caregiver (S Scotland, 33, F, White)		
Infant development		
"Because it is good for my daughter to play with other children and for me to socialise with other parents”<br>Parent/Caregiver (Mid Wales, 39, M)	“Cognitive development, memory resources, interaction, fun and learning through play and music. Community spirit and developing friendships”<br>Provider (Music and sensory experience, 43, F, Any Other Mixed/Multiple)	"The aim is to provide a class that is more than just singing nursery rhymes. Giving children the chance to experience a real live instrument and help their development using musical techniques"<br>Provider (Music education, Yorkshire and the Humber, 35, F, White)
Music development		
"To support the family with musical ideas to help baby's musical and non-musical development"<br>Provider (Music facilitator, 28, White)		
For baby in utero		
" When I was pregnant with my little girl, her movements had stopped/slowed down. I had to attend hospital to be monitored. After half an hour, she had moved twice. As soon as they started to play music in my room, she kept kicking and turning. I was so relieved. [...] I was told my baby liked music as she moved loads when it happened. So was told to try and play music as much as I can"<br>Parent/Caregiver (Yorkshire and the Humber, 33, F, White)		
**Parent/caregiver-Infant bonding**		
“It was lovely to spend time bonding with my child and see other children having fun”<br>Parent/Caregiver (W Midlands, 31, F, White)	“For parents and babies to enjoy in the moment. To encourage parents to "tune in" to their babies"<br>Provider (Community-based music, E Midlands, 55, F, White).	
**Negative experiences**		
“I got joy from seeing her so happy and involved but really these sessions are boring for parents”<br>Parent/Caregiver (N Ireland, 36, F, White)	“I remember feeling a bit embarrassed, but my baby loved it which is the main thing …It was out of my comfort zone and I felt embarrassed”<br>Parent/Caregiver (SW England, 28, F, White)	
